# Influence of an external electric field on the catalytic CO oxidation of nanoscaled CeO_2_ and its La^3+^ and Gd^3+^ congeners

**DOI:** 10.3762/bjnano.17.70

**Published:** 2026-08-04

**Authors:** Parastoo Jamshidi, Silvio Heinschke, Jörg J Schneider

**Affiliations:** 1 Eduard-Zintl-Institut für Anorganische und Physikalische Chemie, Technische Universität Darmstadt, Peter-Grünberg-Str. 12, Darmstadt, Germany

**Keywords:** cerium oxide, CO oxidation, defect structure, doped cerium oxide, electric potential

## Abstract

Herein, we investigate how aliovalent doping and application of an external electrical field modulates the catalytic activity of nanoscale ceria (*n*CeO_2_) towards CO oxidation. Gd^3+^ and La^3+^ were doped into the *n*CeO_2_ lattice. Such doping influences the concentration and mobility of oxygen vacancies, which are promoting CO oxidation to CO_2_. When applying an electric potential, charge transport within *n*CeO_2_ is modulated. Our results show that both dopants Gd^3+^ and La^3+^ lead to an increasing V_O_ concentration, whereas the impact of the applied potential on the oxidation behaviour varies with catalyst composition. The applied electric potential markedly improves the activity of undoped *n*CeO_2_ and 5% Gd^3+^-doped *n*CeO_2_; however, it causes no significant change in CO oxidation capability in 2.5% Gd^3+^-doped and La^3+^-doped samples. These findings can be understood considering the applied potential and its influence on the polaron mobility and V_O_ arrangement, which are controlled by the underlying oxidic defect structure.

## Introduction

Catalytic CO oxidation is a straightforward approach to reduce CO levels. A variety of catalysts have been synthesized and applied to improve the efficiency of CO oxidation [1]. The potential applicability of CeO_2_ in this reaction as well as in photocatalysis, gas sensing, solar cells, electrocatalysis, and as ultraviolet absorbent has been investigated extensively [2]. CeO_2_ crystallizes in the fluorite crystal structure in which each Ce^4+^ is coordinated by eight oxygen anions in a face-centered cubic (fcc) lattice [3]. Characteristically, ceria often shows a mixed valence situation in which both Ce^4+^ and Ce^3+^ exist [4]. Although there are numerous works that have used this mixed valence situation in CeO_2_ to influence catalytic properties, current studies still lack investigations of an external electric field to enhance CO oxidation in nanoscaled CeO_2_ (*n*CeO_2_) in the absence of any precious metals. An external electric field can modify a material’s crystal structure and lattice parameters; for example, it drives charge and mass separation and transport and influences heat transfer occurring in the bulk and at surfaces of dielectrics, piezoelectrics, and electrostrictive materials [5–10]. The electric-field-assisted catalytic decomposition over Pt/CeO_2_ enables the conversion of methanethiol to H_2_S even at low temperatures. An external electric field enhances the formation rates of CH_4_ and H_2_S [11]. Applying a +10 V potential to Au and Pd nanocatalysts increases the CO oxidation rate by 28% and 14%, respectively [12–13]. Under positive bias, Pt (18–54%) and Pd (14–42%) exhibited markedly higher activity than under negative bias (Pt: 4–8% and Pd: 4–12%) [14–15]. The catalytic decomposition of ammonia over organoboron nanoparticles was also examined under an external electric potential. Applying positive or negative potential allowed the decomposition rate to be tuned between +26% and −37% relative to the zero-bias condition [16], indicating that the sign of the applied potential also affects the overall reaction rate. Very recently, the influence of an electric field on the oxidation of 1,2-dichloroethane on CeO_2_ and doped WO*_x_*/CeO_2_ has proven that a coupled mechanism of electronic conduction and lattice oxygen migration can be accelerated applying an external electrical field [17].

The oxygen deficiency of CeO_2_ can be manipulated in a controlled manner by changing temperature and reduction conditions. Also elemental doping of the underlying fcc solid-state structure may create nonstoichiometric species, for example, Ce_1−_*_x_*M*_x_*O_2−δ_ (M = metal) [3,16]. Under these conditions oxygen vacancies (V_O_) are formed. The incorporated dopant elements thus allow one to modify the coordination environment in the CeO_2_ lattice by creating oxygen vacancies, decreasing the energy of V_O_ formation, and improving charge migration [18–20]. When an V_O_ is created in doped ceria, one of the unpaired electrons from the vacant oxygen site fills the hole left behind, and the remaining second electron reduces a tetravalent cerium atom. This contrasts with the V_O_ formation in the undoped ceria surface where two Ce^4+^ atoms are reduced. Doping ceria with various cations influences this general mechanism and changes the energy of the defects and their accessibility, as well as migration energy and migration pathways of mobile ions within the structure [18,21]. With respect to gas adsorption on the surface of ceria this situation is crucial and should vary between undoped and doped *n*CeO_2_.

For the first time, we report herein on catalytic studies of CO oxidation on *n*CeO_2_ and doped-*n*CeO_2_ under an external low-power electric DC potential. Our results show that Gd^3+^- and La^3+^-doped *n*CeO_2_ influence CO oxidation significantly, but these dopants behave differently when coupling with such low-power electrical fields. In situ FTIR studies were used to follow and monitor the influence of the electrical field by measuring the change in the final gas composition over the headspace of the sample, giving substantial information about the CO oxidation capability on solid *n*CeO_2_ under an external electric field.

## Results and Discussion

### Characterization

A series of *n*CeO_2_, containing Gd^3+^ and La^3+^ (2.5% and 5%), was synthesized by a precipitation method (see Supporting Information File 1). XRD patterns are in accord with JCPDS of 34-0394 and prove the fcc structure (Supporting Information File 1, Figures S5–S9) [22]. To investigate the effect of Gd^3+^ and La^3+^ on the doped *n*CeO_2_, full width at half maximum (β), the values of interplanar spacing (*d*), crystallite size (*D*, Scherrer value), dislocation density (δ value), and number of particles per unit surface area (*N* value) were calculated based on three different measurements; these values are summarized in Tables S3–S5 (Supporting Information File 1). According to the values of δ and ε, doping of Gd^3+^ and La^3+^ causes a higher defect density in the fcc lattice of *n*CeO_2_ as compared to the undoped *n*CeO_2_. The structural integrity of the materials after the CO oxidation process was proven spectroscopically (Supporting Information File 1, Figures S10–S14).

In Raman spectra, the characteristic peak corresponding to the F_2g_ mode at ≈470 cm^−1^ is indicative of the symmetry of the lattice (O_h_, Supporting Information File 1, Figures S15–S19). The fcc structure is thus confirmed again for all materials. No signals for other phases or impurities are observed [23]. The characteristic peaks related to defects, extrinsic V_O_, and intrinsic V_O_, appeared around 250, 540, and 600 cm^−1^, respectively [23–24]. The ceria structure remains stable after the oxidation reaction depicting all characteristic Raman modes; however, additional peaks are observed at wavenumbers higher than 1000 cm^−1^, indicative either of physisorption or chemisorption of gas molecules on the catalyst surface (Supporting Information File 1, Figures S20–S24).

**Figure 1:**
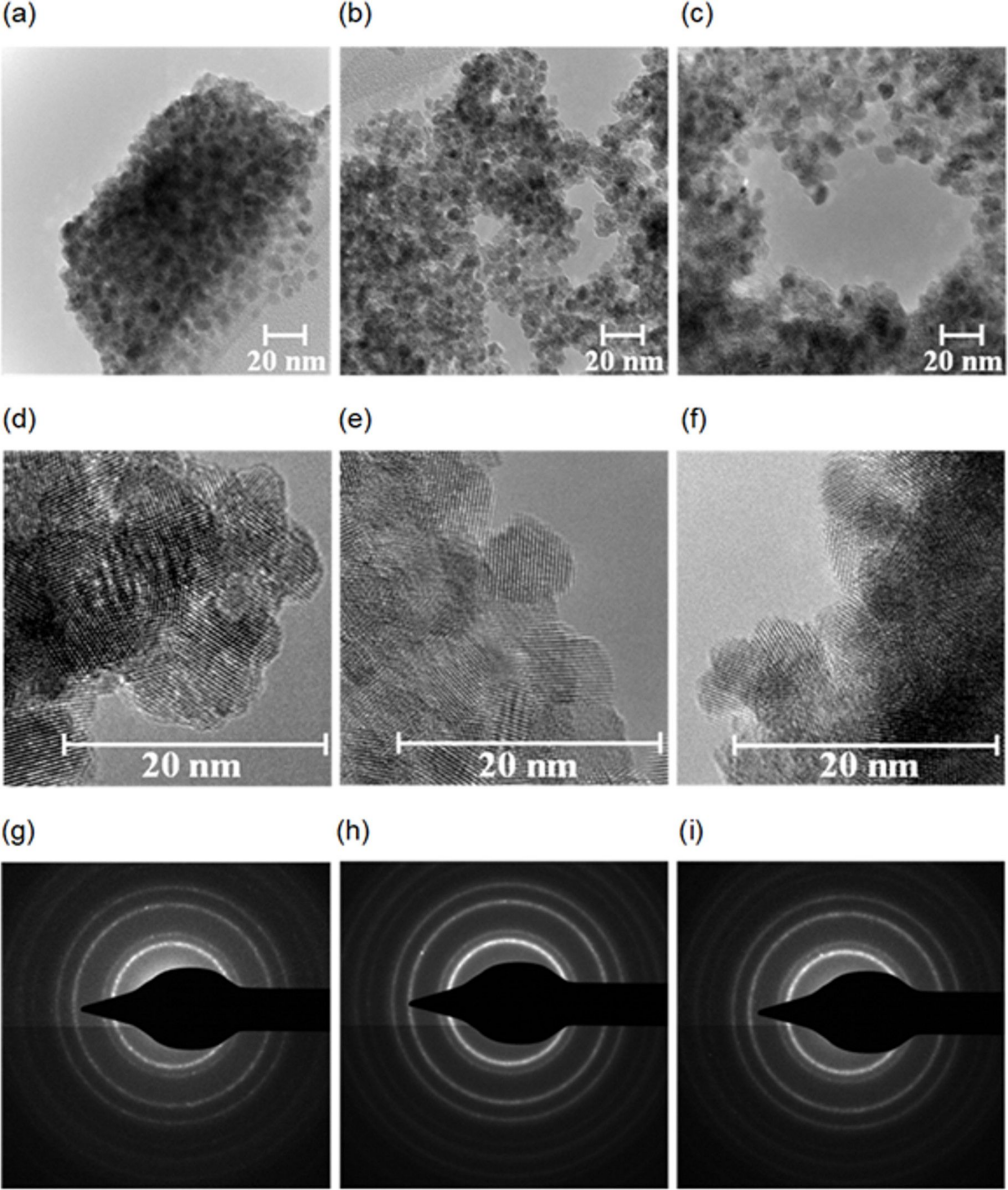
TEM images of (a) *n*CeO_2_, (b) 5%Gd^3+^–*n*CeO_2_ and (c) 5%La^3+^–*n*CeO_2_, HRTEM images of (d) *n*CeO_2_, (e) 5%Gd^3+^–*n*CeO_2_ and (f) 5%La^3+^–*n*CeO_2_, SAED analysis of (g) *n*CeO_2_, (h) 5%Gd^3+^–*n*CeO_2_ and (i) 5%La^3+^–*n*CeO_2_.

All cerium oxide nanoparticles are strongly aggregated and form dense strongly corrugated films with an agglomerated particle size of about 50 nm in scanning electron microscopy (SEM) (Supporting Information File 1, Figure S25). Transmission electron microscopy (TEM) and high-resolution transmission electron microscopy (HRTEM) reveal highly crystalline spherical, slightly faceted *n*CeO_2_ and 5% doped-*n*CeO_2_ with a diameter of around 15 nm (Figure 1a–c). The observed *d*-spacings are characteristic of *n*CeO_2_ (0.31 nm), Gd^3+^–*n*CeO_2_ (0.31 nm), and La^3+^–*n*CeO_2_ (0.29 nm) (Figure 1d–f). This is in accord with the selected-area electron diffraction (SAED) patterns in which pronounced Debye–Scherrer diffraction rings can be assigned to (111), (200), (220), and (311) reflections of cubic CeO_2_ (Figure 1g–i).

The Brunauer–Emmett–Teller theory (BET) surface areas of the samples are determined in the range from 134 to 148 m^2^·g^−1^ (Supporting Information File 1, Table S6).

### CO oxidation on *n*CeO_2_ and doped *n*CeO_2_ without and with applied electrical field

**Figure 2:**
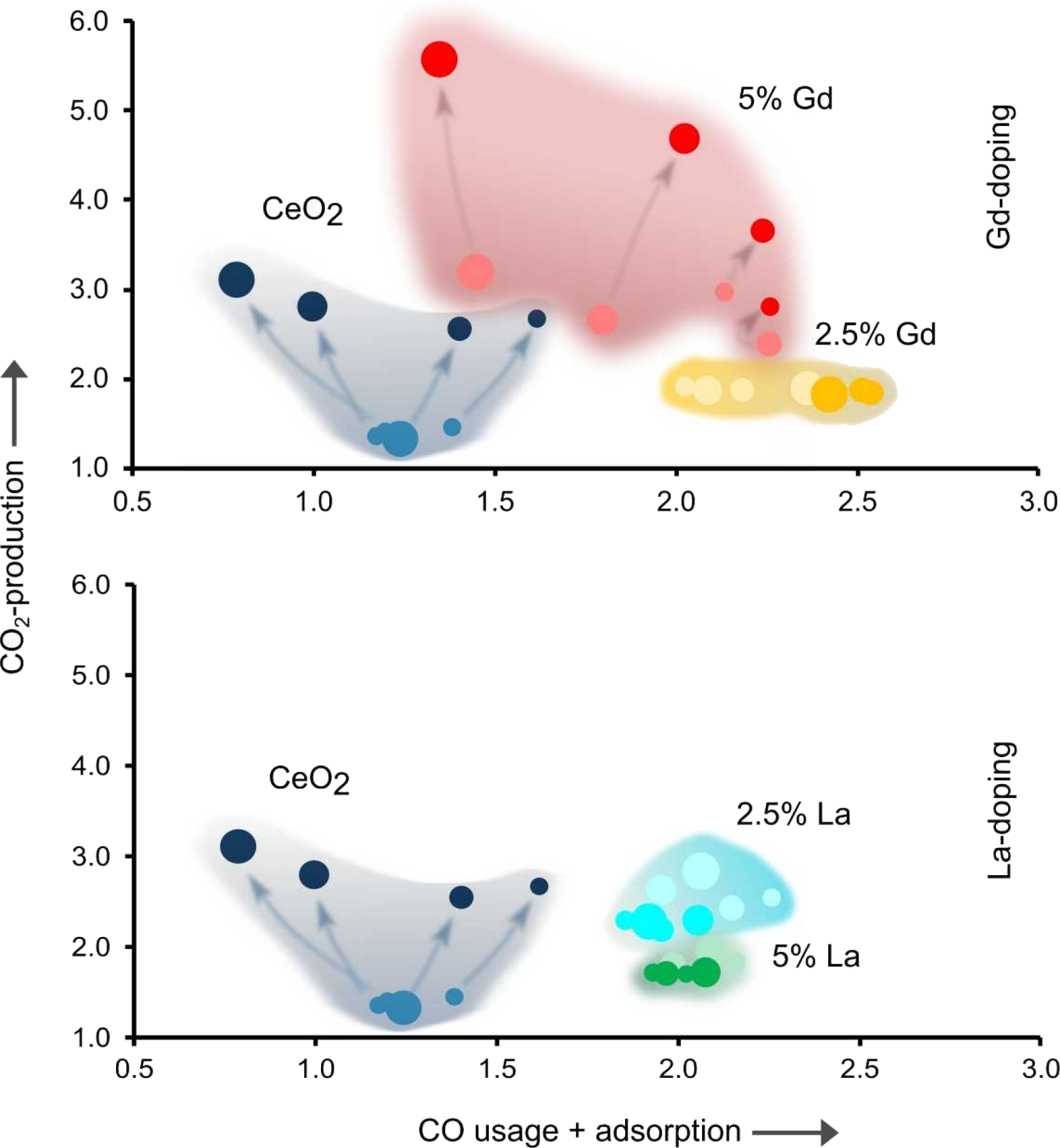
Graphical presentation of the effect of an applied potential (50 V) on CO consumption (*I*_CO_) and CO_2_ production (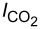) over time (30–120 min) for *n*CeO_2_ and differently doped *n*CeO_2_ catalysts. The arrows indicate the direction of significant changes upon applying potential (50 V). The increasing circle size indicates the progression of reaction time from 30 to 120 min.

Figure 2 illustrates CO consumption (*I*_CO_) and CO_2_ production (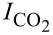) for undoped and doped *n*CeO_2_ catalysts under field-free conditions with an applied potential of 50 V after 30, 60, 90, and 120 min. The increasing circle size indicates the progression of reaction time from 30 to 120 min. The arrows indicate the direction of change in *I*_CO_ and 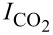 values upon application of the electric field. *I*_CO_ attributes to the CO to CO_2_ conversion and to CO adsorption on the sample (light circles). *n*CeO_2_ showed *I*_CO_ of 1.40 g^−1^ and the doping process increased the value up to 2.00 g^−1^. *I*_CO_ shows a plateau after 60 min from the beginning of the reaction for all different catalysts, under applied potential conditions as well as without electric potential.

Although applying electric potential (50 V) onto *n*CeO_2_ and 5%Gd^3+^–*n*CeO_2_ caused no significant change in *I*_CO_, 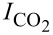 increased substantially (Figure 2 top panel). After 120 min, 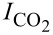 increased from 1.32 to 3.11 g^−1^ for *n*CeO_2_ and from 3.19 to 5.57 g^−1^ for 5%Gd^3+^–*n*CeO_2_, clearly indicating that the electric field promotes CO oxidation, particularly for 5%Gd^3+^–*n*CeO_2_. It thus reveals an increase for CO_2_ production of 170% compared to field-free oxidation conditions. For 2.5%Gd^3+^–*n*CeO_2_, a higher CO uptake was again observed under 50 V; however, CO_2_ production remained approximately constant (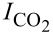 varied from 1.72 to 1.92 g^−1^) (Figure 2 lower panel). Interestingly and in contrast to the Gd^3+^-doped samples, applying the same electric potential affects the La^3+^-doped *n*CeO_2_ samples unexpectedly differently. There was no change in *I*_CO_ under the same electric field for the La^3+^-doped samples (2.5%La^3+^–*n*CeO_2_: from 2.26 to 2.05 g^−1^ and 5%La^3+^–*n*CeO_2_: from 2.15 to 2.07 g^−1^). La^3+^-doped samples showed only small changes in CO_2_ production under field-free and under applied-field conditions. 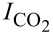 of 2.5%La^3+^–*n*CeO_2_ and 5%La^3+^–*n*CeO_2_ varied from 2.84 g^−1^ to 2.30, 1.97, and 1.72 g^−1^ under field-free vs applied-field conditions, respectively (Figure 2 lower panel and Supporting Information File 1, Tables S7–S12 and Figures S26–S29).

**Figure 3:**
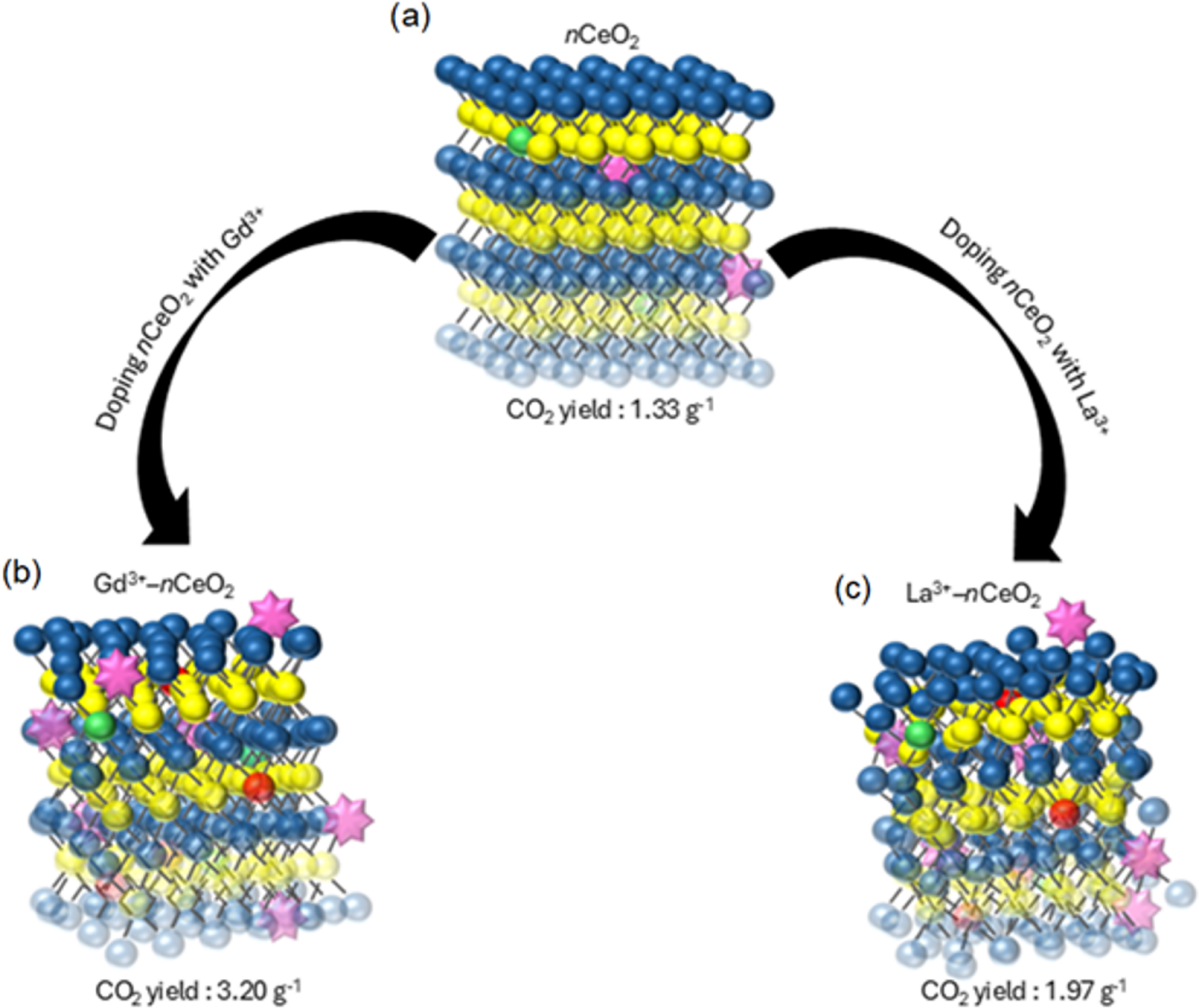
Schematic representation of the proposed defect-related interpretation of CO oxidation over undoped and doped *n*CeO_2_ under field-free conditions. The measured CO_2_ yields are included for comparison. Oxygen vacancies are indicated by pink stars, while Ce^4+^, Ce^3+^, La^3+^/Gd^3+^ dopants, and oxygen are represented by yellow, green, red, and blue spheres, respectively. The fcc layers are shown with decreasing opacity from the surface to the bulk to visualize the near-surface defect distribution. (a) Undoped *n*CeO_2_, showing intrinsic Ce^3+^ formation and associated oxygen vacancies. (b) 5%Gd^3+^–*n*CeO_2_ and (c) 5%La^3+^–*n*CeO_2_, where aliovalent doping increases the oxygen-vacancy population relative to undoped *n*CeO_2_. The schematic highlights the higher near-surface vacancy population in 5%Gd^3+^–*n*CeO_2_, consistent with its higher CO_2_ yield under field-free conditions. The stronger lattice distortion depicted for 5%La^3+^–*n*CeO_2_ is supported by the XRD analysis.

Without applying potential, trivalent rare-earth doping with La^3+^ and Gd^3+^ increases the V_O_ concentration and is expected to increase the population of reduced defect states associated with charge compensation [25–26]. Consistent with this, the doped samples show higher CO_2_ production than undoped *n*CeO_2_ (Figure 3).

**Figure 4:**
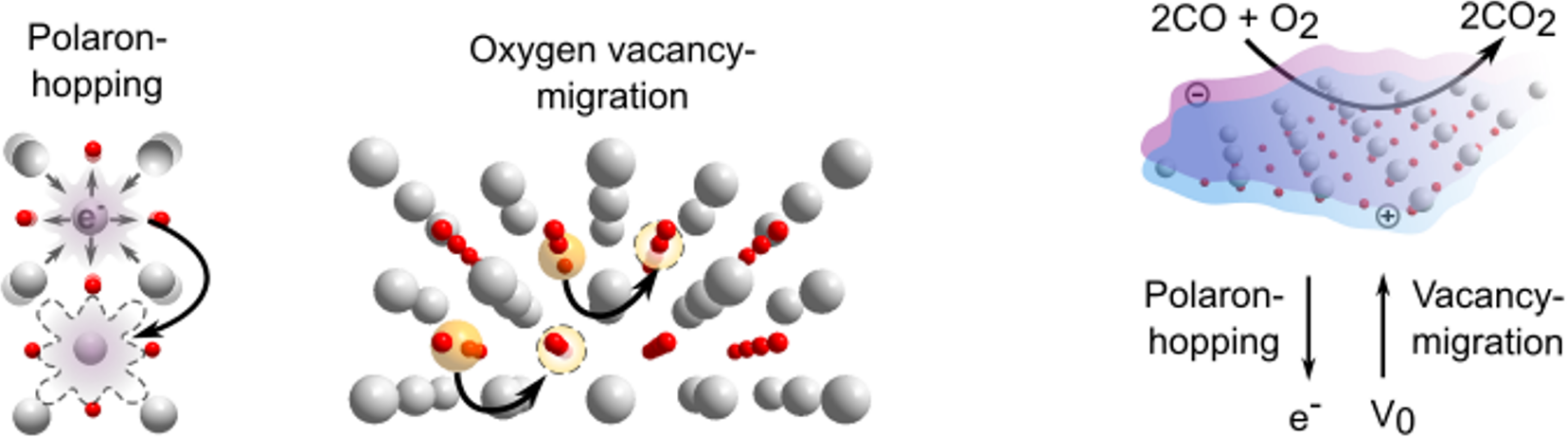
Conceptual schematic representation of possible charge- and defect-transport processes in *n*CeO_2_ under an applied electric field. Grey spheres represent Ce^3+^/Ce^4+^ sites, red spheres represent O^2−^ ions, and yellow spheres indicate V_O_. Small-polaron hopping is illustrated as electron transfer between neighbouring Ce^3+^/Ce^4+^ sites, while V_O_ migration is shown schematically within the fcc lattice. Under an applied electric field, charge redistribution and defect rearrangement may modify the near-surface defect environment and contribute to the observed field-assisted CO oxidation response. The scheme is intended as a conceptual mechanistic framework rather than direct experimental evidence for quantified polaron or V_O_ transport in this work.

External electrical bias has been reported to influence catalytic reactions on solid surfaces by modifying interfacial charge distribution and coupled electronic and ionic defect processes, particularly near surfaces and electrode/oxide interfaces [16,27–30]. In ceria, these processes are closely connected to the Ce^4+^/Ce^3+^ redox couple and V_O_ defect chemistry. A small polaron in *n*CeO_2_ can be described as a localized electron associated with a Ce^3+^ 4f state, and electronic charge transport is commonly discussed in terms of small-polaron hopping between neighbouring cerium sites (Figure 4, left panel) [31–34]. In parallel, oxygen vacancies may rearrange within the fcc ceria lattice under suitable conditions (Figure 4, middle panel) [35]. In the present work, these processes are used as a phenomenological framework to interpret the observed bias-dependent CO oxidation response (Figure 4, right panel).

**Figure 5:**
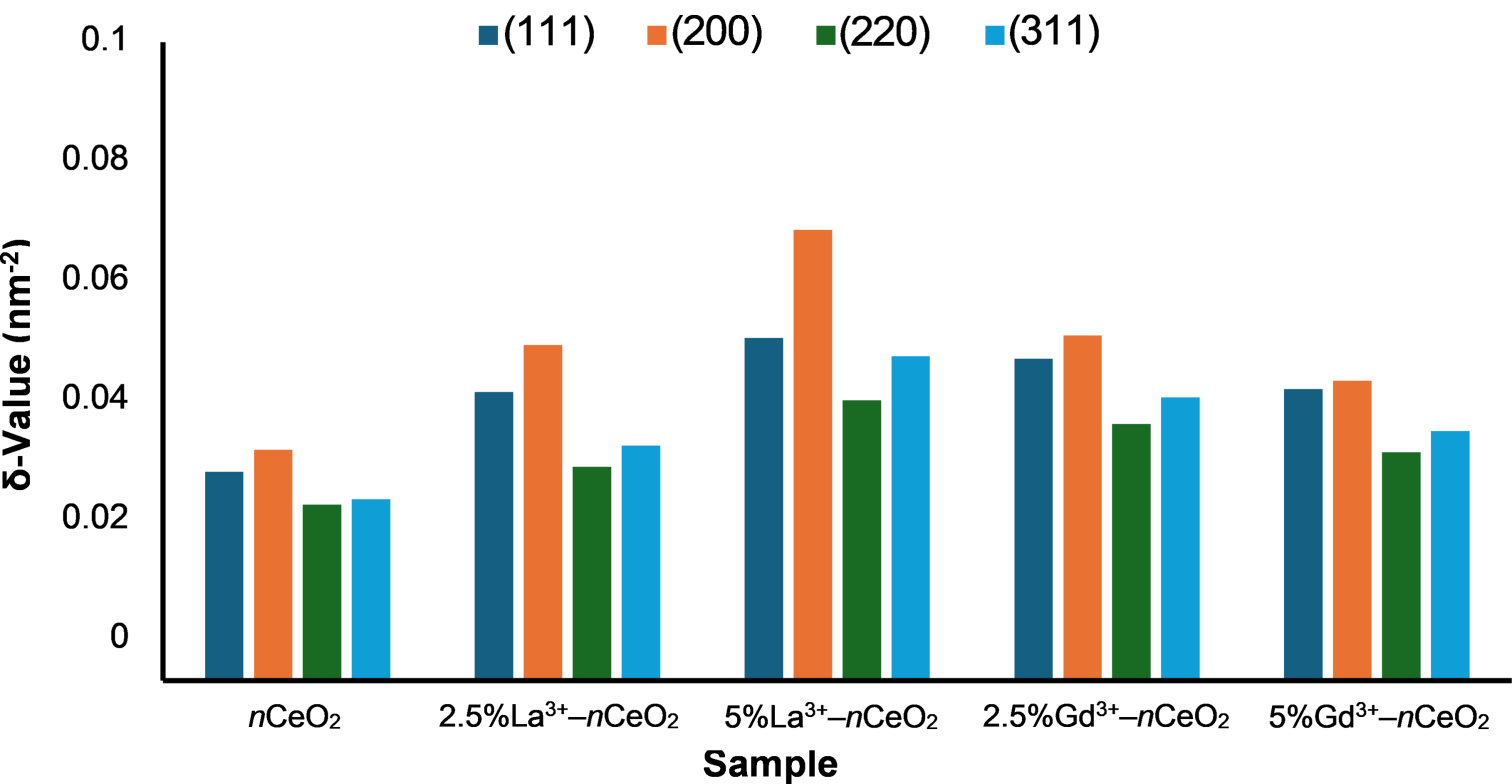
The δ values extracted from the XRD patterns of *n*CeO_2_ and doped *n*CeO_2_ samples with dopant concentrations of 2.5% and 5%, evaluated from the (111), (200), (220), and (311) reflections. The increase in δ values upon doping indicates enhanced lattice distortion relative to undoped *n*CeO_2_, particularly for 5%La^3+^–*n*CeO_2_.

A pronounced synergistic enhancement was also observed for 5%Gd^3+^–*n*CeO_2_, whereas the La^3+^-doped *n*CeO_2_ sample showed no measurable bias-induced improvement in catalytic activity. This dopant-dependent response can be rationalized in terms of the different lattice strain and defect interactions introduced by Gd^3+^ and La^3+^. Regarding Ce^4+^ (0.97 Å), Gd^3+^ (1.053 Å) is closer in ionic radius than La^3+^ (1.16 Å); it is therefore expected to perturb the fluorite lattice less strongly. Consistent with this interpretation, the δ values extracted for 5%La^3+^–*n*CeO_2_ from the (111), (200), (220), and (311) reflections are higher than those obtained for *n*CeO_2_ and 5%Gd^3+^–*n*CeO_2_ (Figure 5), indicating stronger lattice distortion.

**Figure 6:**
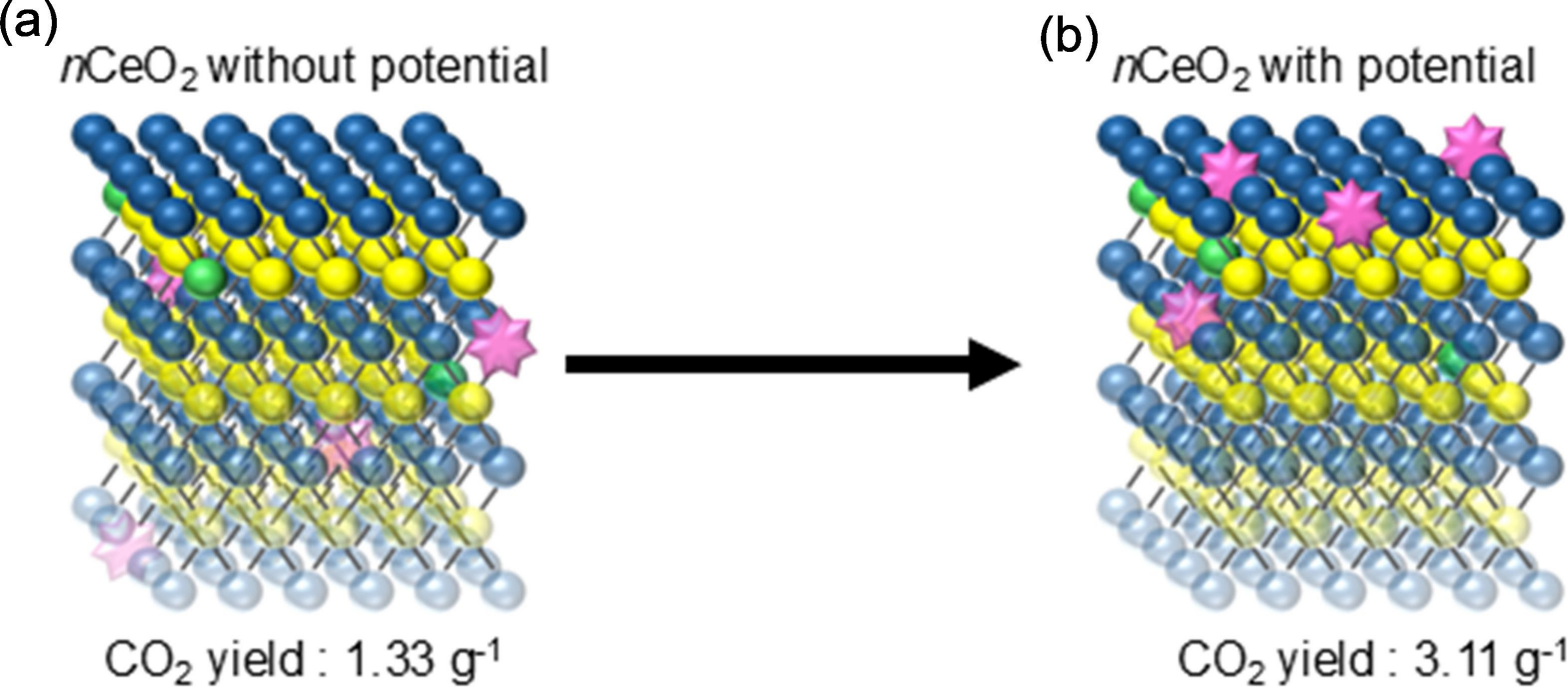
Schematic representation of undoped *n*CeO_2_ under field-free conditions and under an applied external potential. Ce^4+^ (yellow), Ce^3+^ (green), O^2−^ (blue), and oxygen vacancies (pink) are represented. The atomic layers are shown with decreasing opacity from the surface to the bulk to suggest depth. (a) *n*CeO_2_ under field-free conditions, illustrating a lower near-surface concentration of oxygen vacancies. (b) *n*CeO_2_ under an applied potential, schematically illustrating a possible field-induced charge redistribution and a higher near-surface concentration of oxygen vacancies. The measured CO_2_ yields are included for comparison. This scheme is intended as a conceptual representation of the field-assisted response and not as direct experimental proof of quantified vacancy transport.

Applying an external potential to bare *n*CeO_2_ nearly doubled the apparent CO oxidation efficiency compared with the field-free condition (Figure 6a,b).

The different behaviour of Gd^3+^- and La^3+^-doped *n*CeO_2_ may also be related to their different interactions with oxygen vacancies. Gd^3+^ incorporation is expected to favour the formation of oxygen vacancies while maintaining a comparatively less distorted fluorite lattice. In contrast, the larger La^3+^ ions can promote the formation of more stable defect associates, such as [La^3+^-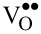-La^3+^] and [La^3+^-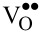-Ce^3+^], which may reduce the ability of oxygen vacancies to rearrange within the lattice or participate in near-surface redox processes [20,36].

**Figure 7:**
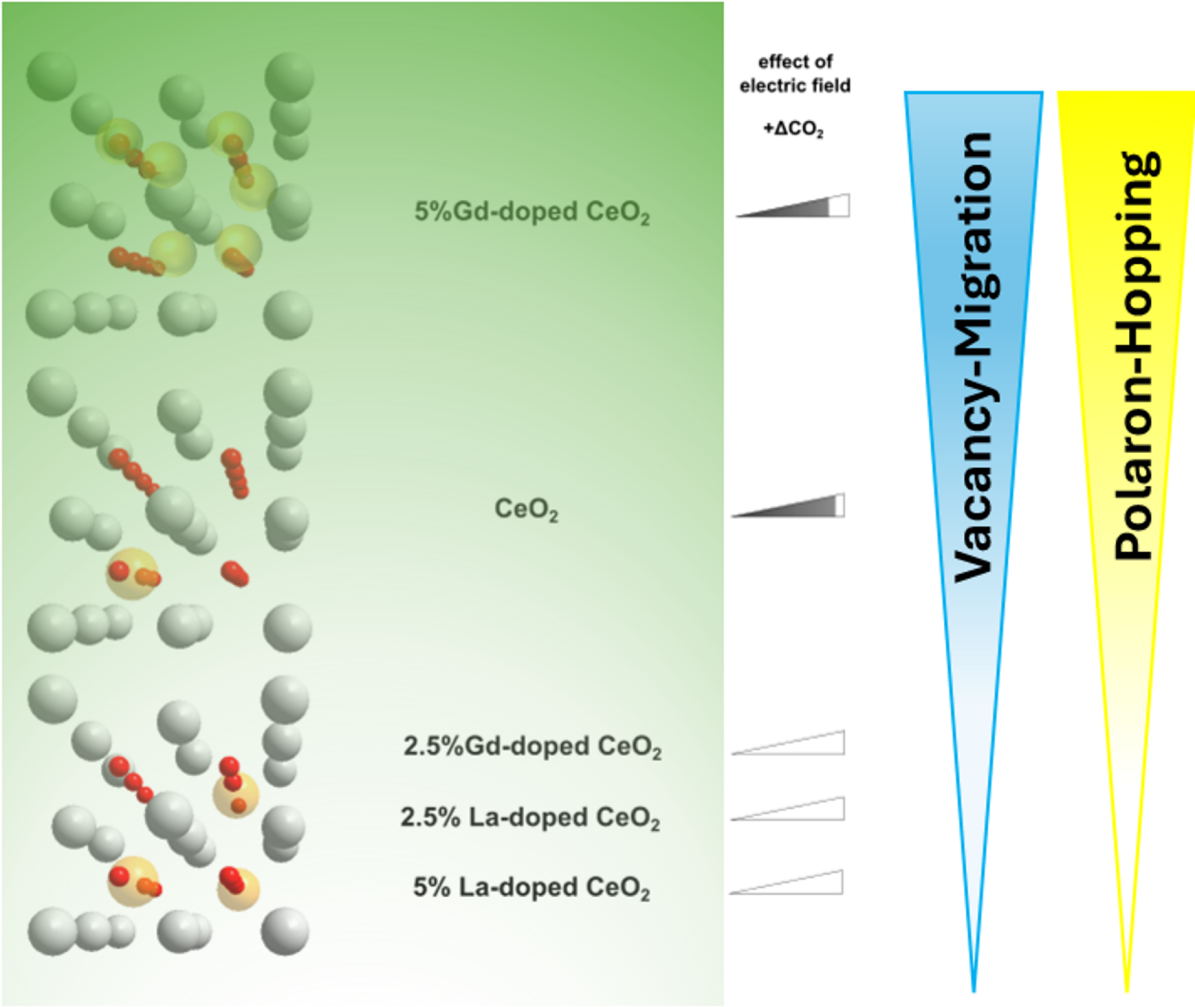
Schematics showing the influence of an external electric field onto *n*CeO_2_ and differently doped *n*CeO_2_ samples. The emerging intense green background from top to bottom indicates the increasing combined effect of vacancy migration and polaron hopping onto the performance of the various catalysts. A synergistic effect of polaron hopping and V_O_ migration is in favour of an enhanced CO oxidation for the low and medium defective samples *n*CeO_2_ and 5%Gd^3+^–*n*CeO_2_. When increasing V_O_ as in 2.5%Gd^3+^–*n*CeO_2_ and 5%La^3+^–*n*CeO_2_, the effect of the external applied field is expected to be compensated in the Gd^3+^-doped samples. La^3+^-doping however even hinders CO oxidation by V_O_ cluster formation.

Under electrical bias, polarization and charge redistribution at grain surfaces may therefore affect the near-surface Ce^3+^/Ce^4+^ population and the availability of oxygen-vacancy-related defect sites involved in CO oxidation. On this basis, the observed CO oxidation behaviour with and without electrical bias suggests that local changes in oxygen-vacancy distribution and associated Ce^3+^/Ce^4+^ redox states may modify the near-surface ionic and electronic environment. The pronounced response of *n*CeO_2_ and 5%Gd^3+^–*n*CeO_2_ to the applied potential is consistent with possible contributions from small-polaron-related charge transport and oxygen-vacancy rearrangement near the surface. In contrast, the weaker response of 2.5%Gd^3+^–*n*CeO_2_ suggests that the dopant concentration may be insufficient to generate a strong coupled defect/electronic response, while the negligible bias-induced improvement observed for La^3+^–*n*CeO_2_ is consistent with a reduced ability of oxygen vacancies to participate in field-assisted surface redox changes, possibly due to the formation of stable La-associated defect clusters (Figure 7).

We emphasize that this mechanistic interpretation remains descriptive and phenomenological. The present data support a correlation between dopant-dependent defect chemistry, lattice distortion, and bias-assisted CO oxidation behaviour, but they do not directly quantify oxygen-vacancy migration or small-polaron hopping during the reaction. Further systematic studies, such as voltage-dependent measurements, operando spectroscopy, oxygen-isotope exchange, conductivity measurements, or theoretical calculations, would be required to verify the microscopic contributions of these processes. Nevertheless, the present results indicate that applying an electrical bias to reducible oxide catalysts can provide a promising strategy for influencing heterogeneous oxidation reactions, even in the absence of precious metal active phases.

## Conclusion

An external electric field can substantially boost room-temperature CO oxidation on nanoscaled ceria by tuning its interfacial defect/redox chemistry. It induces interfacial polarization in ceria based catalysts and boosts CO oxidation under noble-metal-free conditions. The magnitude of the enhancement depends critically on dopant-controlled defect interactions, ion mobility, and electronic polaron transport mechanisms. Undoped *n*CeO_2_ shows the largest activity and thus response to the field, with nearly a twofold increase in apparent CO oxidation efficiency under bias, while 5%Gd^3+^–*n*CeO_2_ exhibits a clear synergistic enhancement consistent with sufficient defect mobility and interfacial responsiveness. In contrast, 2.5%Gd^3+^–*n*CeO_2_ appears insufficient for a strong coupled polaron vacancy response, and La^3+^-doped samples show little to no bias-induced improvement, consistent with stronger defect association/cluster formation that suppresses vacancy participation in sustained field-induced surface redox changes.

## Experimental

### CO to CO_2_ oxidation experiments

The custom-built gas adsorption device consists of sample cell, volume cell, circulation pump, and IR measurement cell, which are interconnected by stainless-steel tubes (6 mm), valves (Swagelok^®^ SS-6P4T-MM-8K), and Swagelok^®^ fittings [37]. The process was conducted under ambient conditions and was controlled by a K-type thermocouple (OMEGA Engineering Inc., NiCr, ±1.1 °C) inside the reactor. The outer and inner electrodes of the sample cell were isolated from each other by tubes (PEEK, 6 mm outer diameter, 1.65 mm inner diameter; 3.6 mm outer diameter, and 1.7 mm inner diameter). A Sorensen^®^ XEL 250 DC Power Supply was used to apply the potential to the sample, where the thermocouple inside and the steel mantle of the sample chamber were used as the inner electrode and the outer electrode, respectively. The measurement cell, composed of a steel tube and sealed by two stainless steel caps (0.9 cm window) using PEEK and two Viton^®^ O-rings each, was placed inside a FTIR spectrometer (Thermo Fisher^®^-Nicolet iS5 with ID1 Transmission Accessory; Software Omnic 9.2.106). Each cap held 1.5 mm thick quartz lenses. The FTIR spectrometer was used to record the gas mixture spectra. A circulation pump (GK-M-12/02) and a pressure transducer (PAA33X-V-30 (0–30 bar), accuracy of 0.05% and resolution of 0.002%) were purchased from Gardner Denver Thomas GmbH, Germany and Omega Newport electronics GmbH, Germany, respectively.

**Figure 8:**
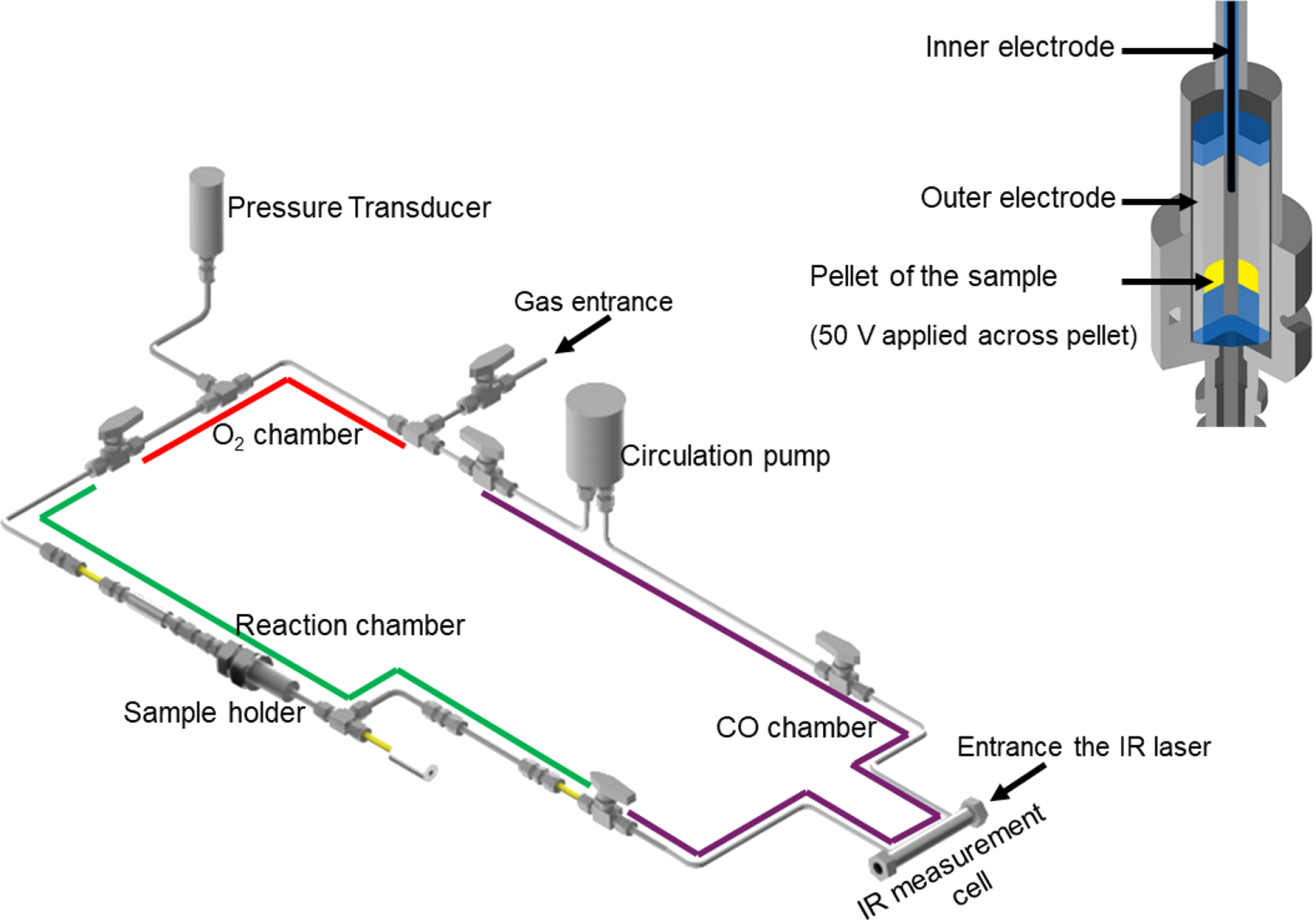
Schematic representation of the gas adsorption/IR measurement setup. The device consists of valve-separated O_2_, CO, and sample/reaction chambers, allowing for independent evacuation, gas dosing, equilibration, mixing, and controlled exposure of the catalyst pellet to the gas mixture. The pressure was monitored using a pressure transducer, and CO/O_2_ mixing was assisted by a circulation pump. The inset shows the sample-cell configuration, including the inner and outer electrodes and the pressed catalyst pellet. The external voltage was applied across the pressed catalyst pellet through the inner and outer electrodes. The corresponding gas dosing and exposure sequence is summarized in Supporting Information File 1. The graphic was created with Solid Edge. This content is not subject to CC BY 4.0.

The FTIR spectra were recorded from 400 to 4000 cm^−1^, considering integration in the ranges from 2143 to 2231 cm^−1^ and from 2283 to 2389 cm^−1^, related to CO and CO_2_ signals, respectively. The integration values for the cell under vacuum and for each measurement were calculated by OMNIC software. The schematic setup of the device is shown in Figure 8.

### Oxidation reaction process

The system consisted of three valve-separated compartments, namely, an O_2_ chamber (≈17 cm^3^), a CO chamber (≈30 cm^3^), and a sample chamber (≈18 cm^3^). These compartments are indicated by different colours in Figure 8. This configuration allowed the gases to be introduced, isolated, mixed, and exposed to the catalyst in a controlled manner. The pressure in the system was monitored using a pressure transducer, whose output signal was read with a multimeter.

The pressed catalyst pellet was placed in the sample chamber at the end of the cell. The catalyst amounts are shown in Supporting Information File 1, Table S3. Before gas dosing, the sample chamber was heated to approximately 120 °C under vacuum for 15 h to remove weakly adsorbed species, including adsorbed gases, water, and possible residual ammonia, from the sample surface and the cell environment. After this pre-treatment, the system was kept under vacuum and cooled to room temperature before gas dosing. During the vacuum pre-treatment and before gas dosing, the pressure reading was close to zero within the resolution of the pressure-monitoring system. The vacuum spectrum was recorded in this step.

The CO-containing gas mixture, CO/N_2_ = 0.1/99.9, Air Liquide, Germany, was first introduced into the CO chamber up to a pressure of 1.5 bar. After an equilibration period of 15 min and reaching a nearly constant pressure signal, the CO chamber (purple region in Figure 8) was isolated by closing the valve between the CO and O_2_ chambers. The O_2_ chamber was then evacuated for 10 min to remove residual gas before O_2_ was introduced up to a pressure of 1.5 bar. The O_2_ chamber is shown as the red region in Figure 8. After a further equilibration period of 15 min, the reference CO spectrum was recorded before opening the valve between the CO and O_2_ chambers, while the two gases were still kept in separate compartments.

Subsequently, the valve between the CO and O_2_ chambers was opened, and the gases were mixed using a circulation pump. After 15 min of mixing/equilibration, the spectrum of the CO/O_2_ gas mixture was recorded. The upstream and downstream valves of the sample holder were then opened to expose the catalyst pellet to the gas mixture (the green region in Figure 8). Due to expansion, the pressure of the gas mixture decreased to around 1 bar. Spectra were recorded after 10, 30, 60, 90, and 120 min of contact between the gas mixture and the catalyst.

For the experiments performed under an external electric field, the same gas dosing, mixing, and equilibration procedure was followed. A DC voltage of 50 V was applied using a potentiostat in a two-electrode configuration, with the inner and outer electrodes of the cell serving as the two electrodes. The applied voltage (50 V) generated a DC electric field across the catalyst-containing region. The voltage was applied before opening the upstream and downstream valves of the sample holder and was maintained throughout the full reaction period of 120 min. Spectra were recorded after 10, 30, 60, 90, and 120 min of exposure to the CO/O_2_ gas mixture.

The circulation pump was switched off during spectral acquisition. After the final spectrum was collected, the system was evacuated again. A process-flow diagram summarizing the evacuation, gas dosing, equilibration, CO/O_2_ mixing, and exposure of the gas mixture to the catalyst pellet is provided in Supporting Information File 1. For details about the calculation of the gas compositions, see Supporting Information File 1.

## Supporting Information

Supporting Information File contains more information about synthesis, characterization and raw data values of the measurements.

File 1 Additional information.

## Data Availability

All data that supports the findings of this study is available in the published article and/or the supporting information of this article.
