# Supplementary material for: Influence of an external electric field on the catalytic CO oxidation of nanoscaled CeO2 and its La3+ and Gd3+ congeners
Source: Beilstein J Nanotechnol. 2026 Aug 4;17:1028–38. doi: 10.3762/bjnano.17.70 (PMC13454883; doi:10.3762/bjnano.17.70)
Supplement: Supplementary file 1 [file Beilstein_J_Nanotechnol-17-1028-s001.pdf]

BEILSTEIN JOURNAL OF NANOTECHNOLOGY

Supporting Information

for

Influence of an external electric field on the catalytic CO oxidation of nanoscaled CeO_2 and its La^{3+} and Gd^{3+} congeners

Parastoo Jamshidi, Silvio Heinschke and Jörg J. Schneider

Beilstein J. Nanotechnol. **2026,** *17,* 1028–1038. doi:10.3762/bjnano.17.70

Additional information

License and Terms: This is a supporting information file under the terms of the Creative Commons Attribution License (https://creativecommons.org/licenses/by/4.0). Please note that the reuse, redistribution and reproduction in particular requires that the author(s) and source are credited and that individual graphics may be subject to special legal provisions.
The license is subject to the *Beilstein Journal of Nanotechnology* terms and conditions: (https://www.beilstein-journals.org/bjnano/terms)

Experimental

Materials and reagents

Supporting information text Ammonia solution (30%), cerium(III)nitrate hexahydrate, lanthan(III)-nitrat Hexahydrat and gadolinium(III)nitrate pentahydrate were provided by (Ceel, Belgium), thermoscientific (www.thermofisher.com; Massachusetts, US.) and Alfa Aesar (Karlsruhe, Germany) companies. HPLC water, provided by fisher chemical company (Massachusetts, US.), was used. All chemicals were of analytical reagent grade and were used without any extra purification.

Instruments for characterization

X-ray diffraction (XRD; Model: Rigaku Miniflex 600@40 kV 15 mA diffractometer using Cu-Kα radiation (λ = 1.541 Å)) and Raman spectroscopy (Model: DXR3) characterized the crystallinity, changing the lattice and studying the defect. Scanning electron microscopy (SEM) was applied to picture the morphology of the materials. For high resolution transmission electron microscopy (HRTEM) investigations, a FEI Tecnai G2 F20 with an operating voltage of 200 keV was used.

Synthesis of the materials

Synthesis of $n\mathrm{CeO_2}$

Cerium (III) nitrate hexahydrate (2.0 g) was solved in distilled water (100 mL). Ammonia (120 mL) and distilled water (30 mL) were mixed and added drop by drop to the previous solution. The suspension kept stirring for 15 hours, then centrifuged, washed two times with distilled water and dried at 80 °C. No further thermal treatment was applied to the samples.

Synthesis of doped *n*CeO_2

Specific amounts of lanthanum (III) nitrate hexahydrate and gadolinium (III) nitrate pentahydrate were solved in a solution of cerium (III) nitrate hexahydrate (2.0 g) and distilled water (100 mL). Ammonia (120 mL) and distilled water (30 mL) were mixed and added drop by drop to the previous solution. The suspension kept stirring for 15 hours. Gd^{3+}–*n*CeO_2 and La^{3+}–*n*CeO_2 samples were centrifuged, washed two times with distilled water and dried at 80 °C. No further thermal treatment was applied to the samples.

Calibration

The calibration of the system was explained in details in previous article of this group [1]. Also, for finding any error pressure transducer measurements, N_2 was purged into CO and O_2 chamber and after equilibrium, it was released to the empty sample chamber. The initial pressure and pressure after 120 minutes are provided in Table S1. The average ratio is 1.2587 ± 0.0017.

Table S1: N_2 expansion test for verification of pressure transducer calibration (*T*: 25 ± 2 °C).

Initial Pressure (bar)	Final Pressure (bar)	Ratio
1.8222	1.4460	1.2601
1.8150	1.4394	1.2609
1.8066	1.4358	1.2582
1.8183	1.4454	1.2579
1.8063	1.4355	1.2583
1.8072	1.4397	1.2552
1.8294	1.4517	1.2601

The amounts of CO and CO_2 in μmol were extracted by putting the integration amounts of spectra in calibration plots for each gas. The capacity of the catalyst for CO-consume and CO_2 production is calculated by Eq.S1 and Eq.S2, respectively.

$$I_{CO} = \frac{initial\ amount\ of\ CO\ (\mu mol) - amount\ of\ CO\ in\ actual\ time\ (\mu mol)}{initial\ amount\ of\ CO\ (\mu mol)} \times \frac{1}{*amount\ of\ catalyst\ (g)}$$

(S1)

$$I_{CO_2} = \frac{mount\ of\ CO_2\ in\ actual\ time\ (\mu mol)}{initial\ amount\ of\ CO\ (\mu mol)} \times \frac{1}{*amount\ of\ catalyst\ (g)} \quad \text{(S2)}$$

In the present work, we adopted a calibration curve for CO gas, which was established over a broad concentration range (Figure S1). For the purposes of our measurements, we restricted the calibration to the narrower interval relevant to the experimental conditions and the expected analyte levels, thereby improving practical resolution within the working range (Figure S2). Minor differences in experimental conditions (e.g., extraction temperature ~ 40 °C) are not expected to affect the interpretation within the selected range and were therefore not treated as a primary variable. In particular, the aim of this paper is a phenomenological interpretation of dopant effects, rather than the reporting of absolute quantities with high metrological precision.

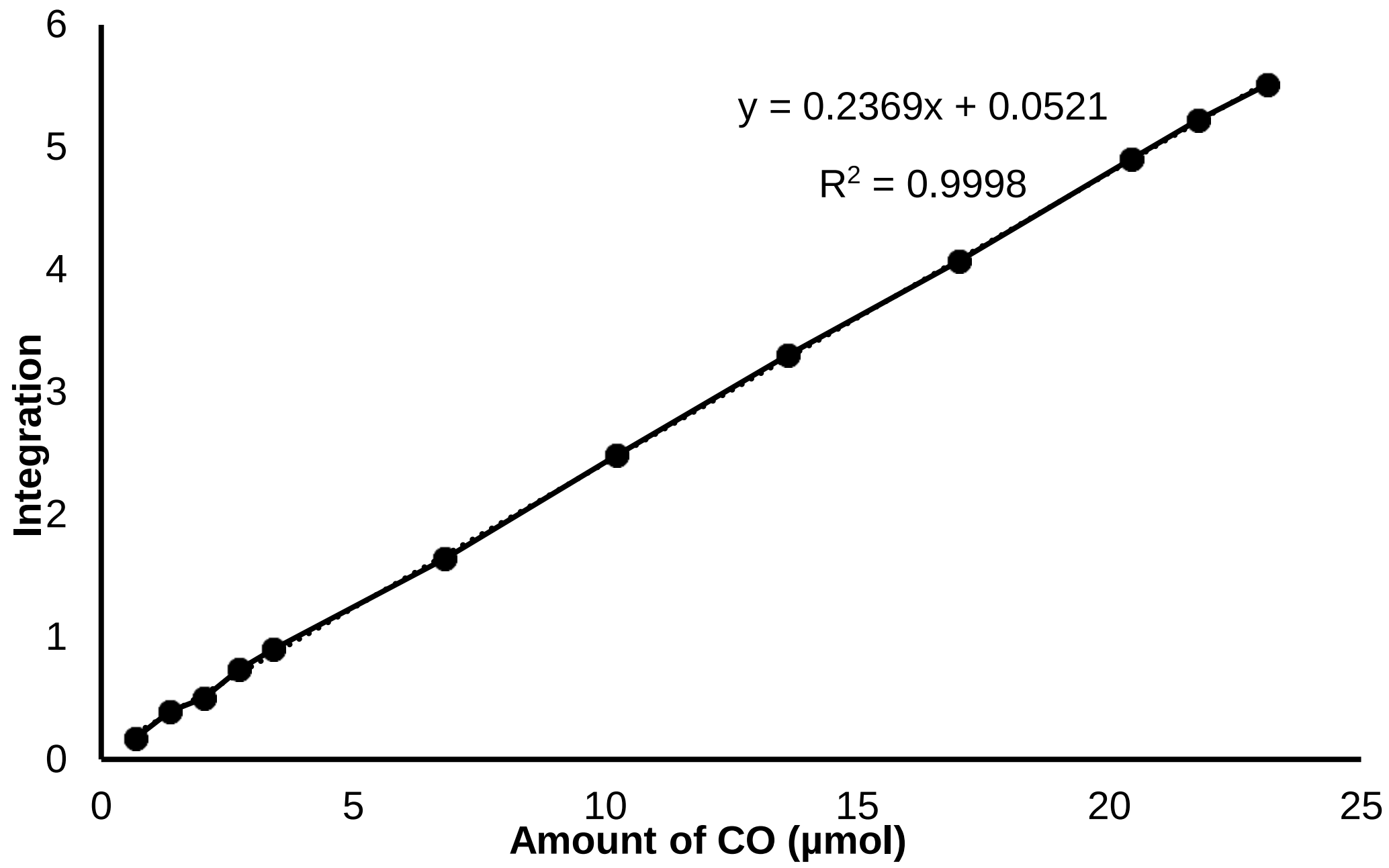

Figure S1: Calibration curve over an extended concentration range of CO gas (T = 40 °C).

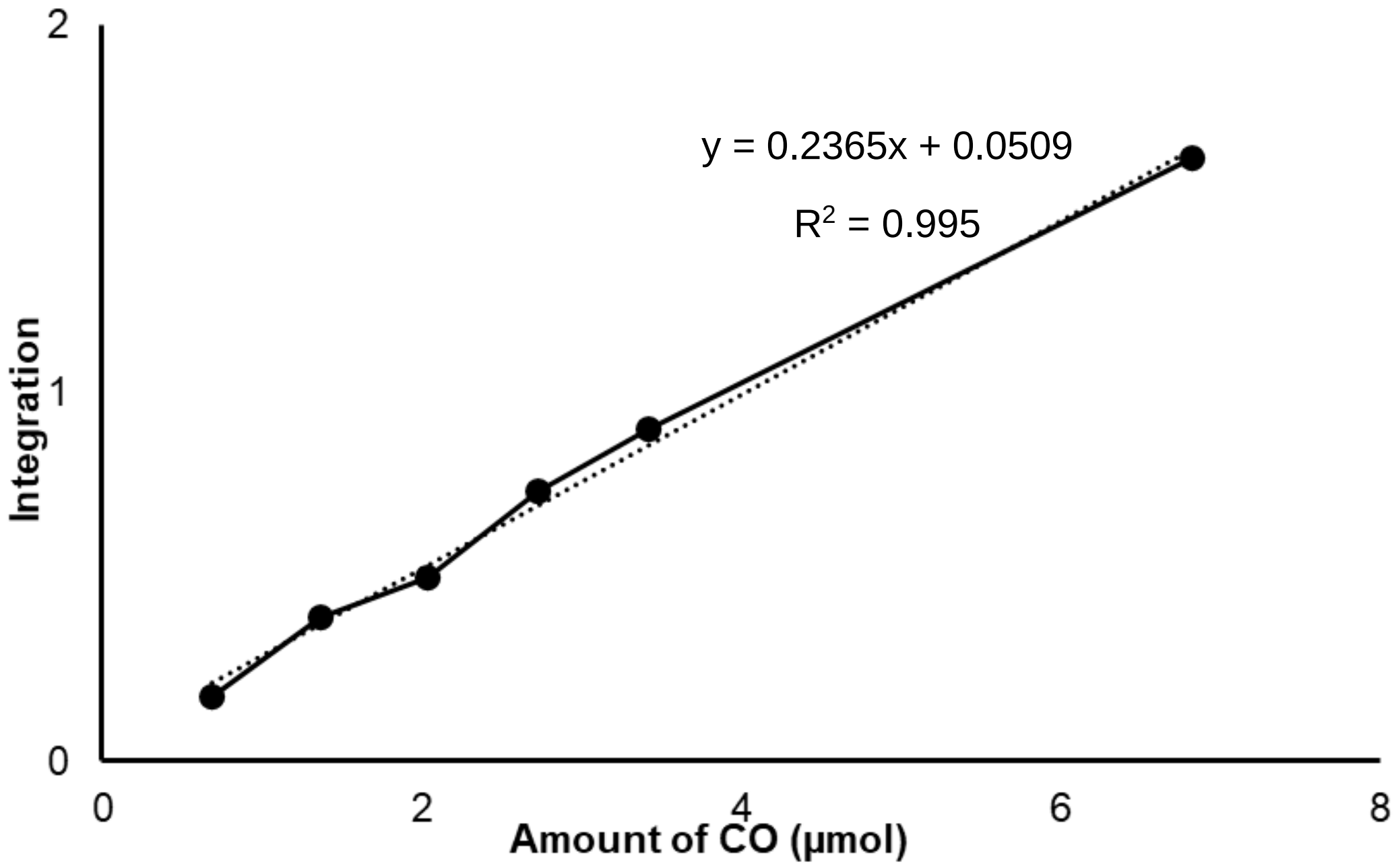

Figure S2: Calibration curve over a restricted concentration range of CO gas (T = 40 °C).

In the present work, we adopted a calibration curve for CO_2 gas, which was established over a broad concentration range (Figure S3). For the purposes of our measurements, we restricted the calibration to the narrower interval relevant to the experimental conditions and the expected analyte levels, thereby improving practical resolution within the working range (Figure S4). Minor differences in experimental conditions (e.g., extraction temperature ~ 40 °C) are not expected to affect the interpretation within the selected range and were therefore not treated as a primary variable. In particular, the aim of this paper is a phenomenological interpretation of dopant effects, rather than the reporting of absolute quantities with high metrological precision.

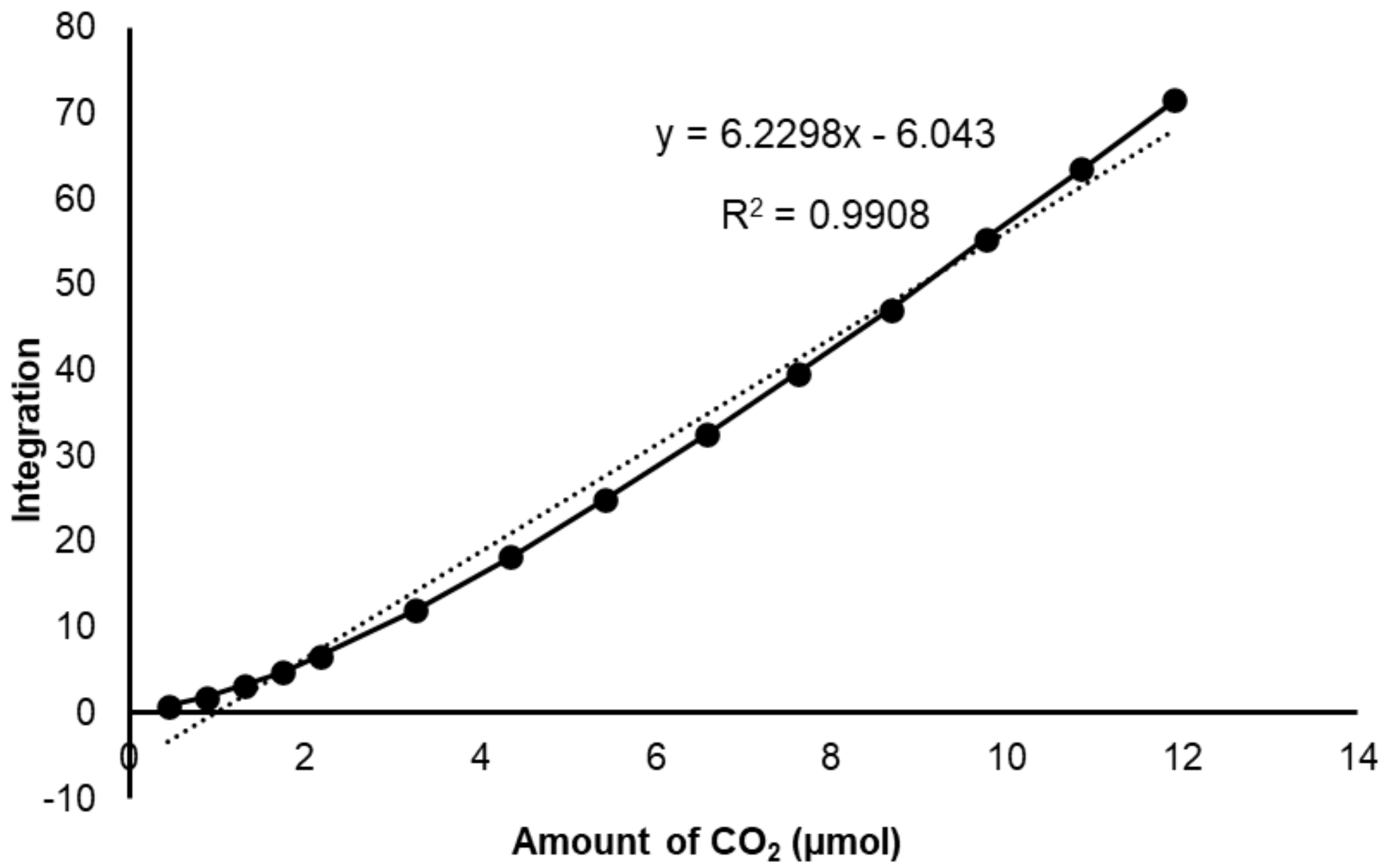

Figure S3: Calibration curve over an extended concentration range of CO_2 gas (T = 40 °C).

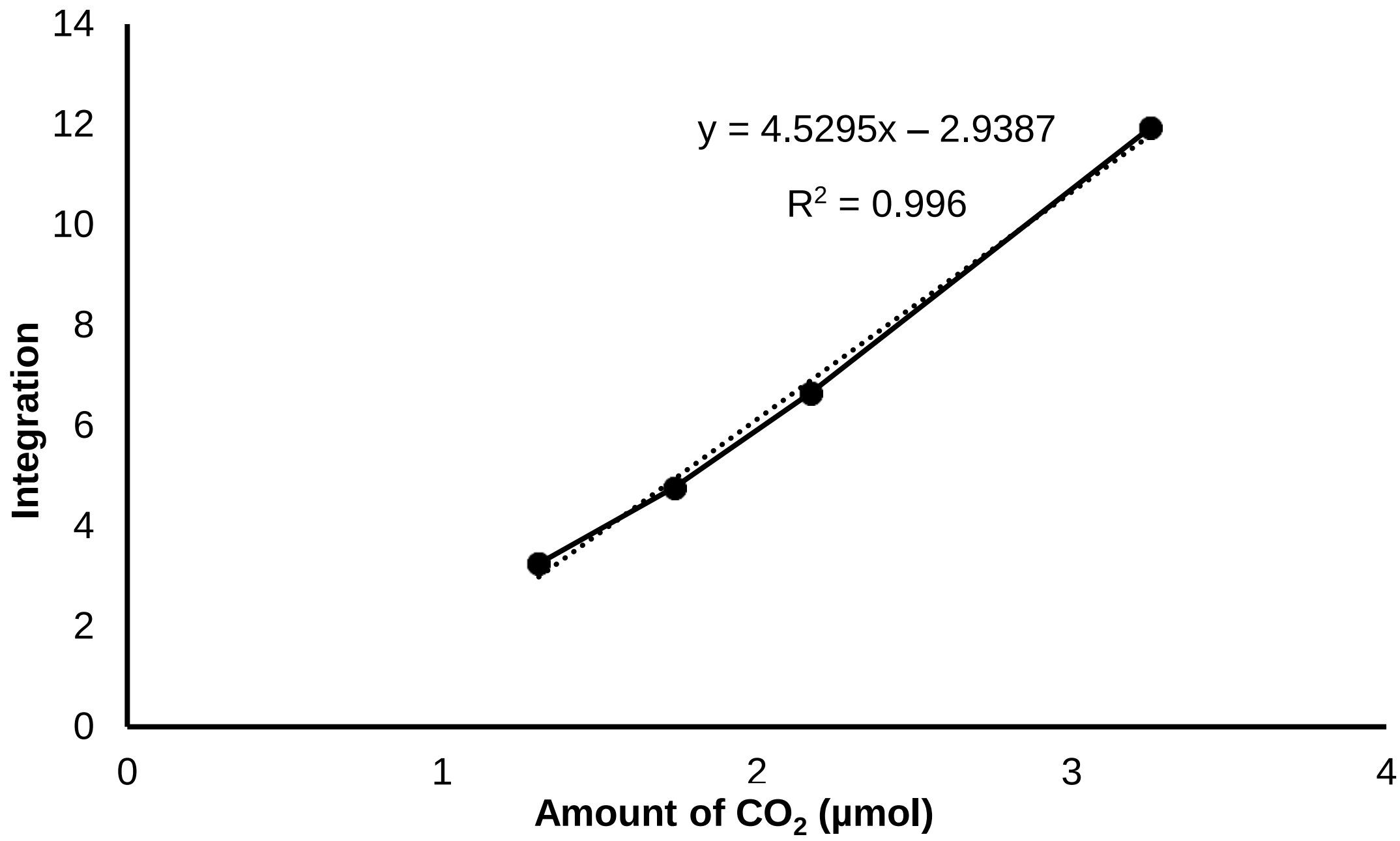

Figure S4: Calibration curve over an extended concentration range of CO_2 gas (T = 40 °C).

The amount of catalyst, used for the reaction, is written in Table S2.

Table S2: Catalysts amount for CO oxidation reaction

**Sample**	**Amount (g)**
nCeO$_2$	0.132
2.5%La^{3+}–nCeO$_2$	0.129
5%La^{3+}–nCeO$_2$	0.122
2.5%Gd^{3+}–nCeO$_2$	0.129
5%Gd^{3+}–nCeO$_2$	0.121

A process-flow summarizing the evacuation, gas dosing, equilibration, CO/O_2 mixing, and exposure of the gas mixture to the catalyst pellet was as following:

Sample pellet placed in sample holder
↓
Vacuum pretreatment at ~120 °C for 15 h
↓
Cooling to room temperature under vacuum
↓
CO dosing to CO chamber, 1.5 bar
↓
CO equilibration, 15 min
↓
O_2 chamber evacuation, 10 min
↓
O_2 dosing to O_2 chamber, 1.5 bar
↓
O_2 equilibration, 15 min
↓
Reference CO spectrum recorded before CO/O_2 mixing
↓
CO and O_2 chambers connected
↓
Gas mixing with circulation pump, 20 min
↓
CO/O_2 gas-mixture spectrum recorded
↓
Valves to sample holder opened
↓
Spectra recorded after 30, 60, 90, and 120 min
↓
System evacuated

Results and Discussion

Characterization of the materials

The Peaks, located at 2θ of 28.30˚, 32.90˚. 47.38˚, 56.16˚, 58.52˚, 68.92˚, 76.76˚ and 78.92˚ are related to the crystal sheets of 111, 200, 220, 311, 222, 400, 331 and 420, respectively [2].

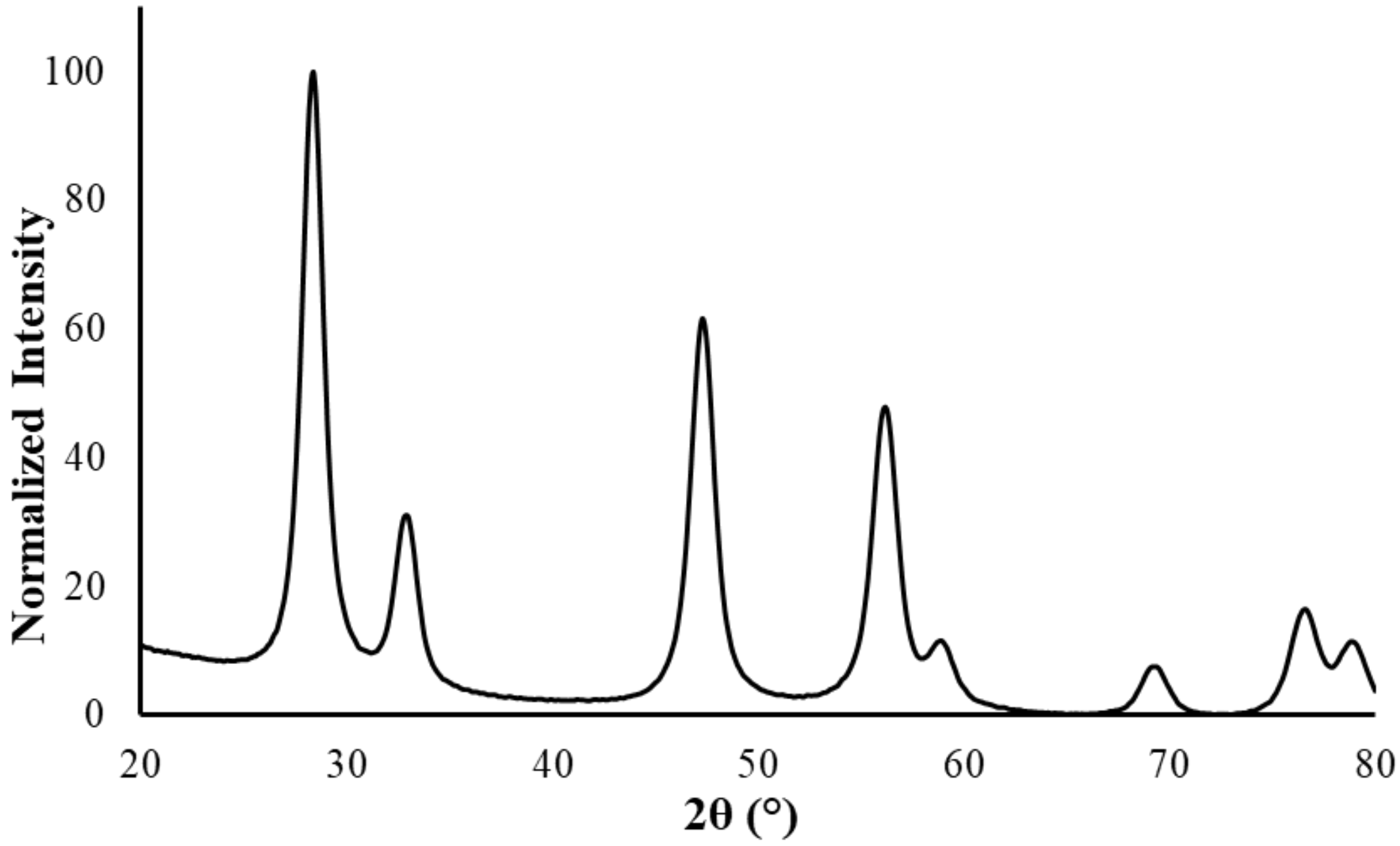

Figure S5: XRD pattern of *n*CeO_2 before the reaction.

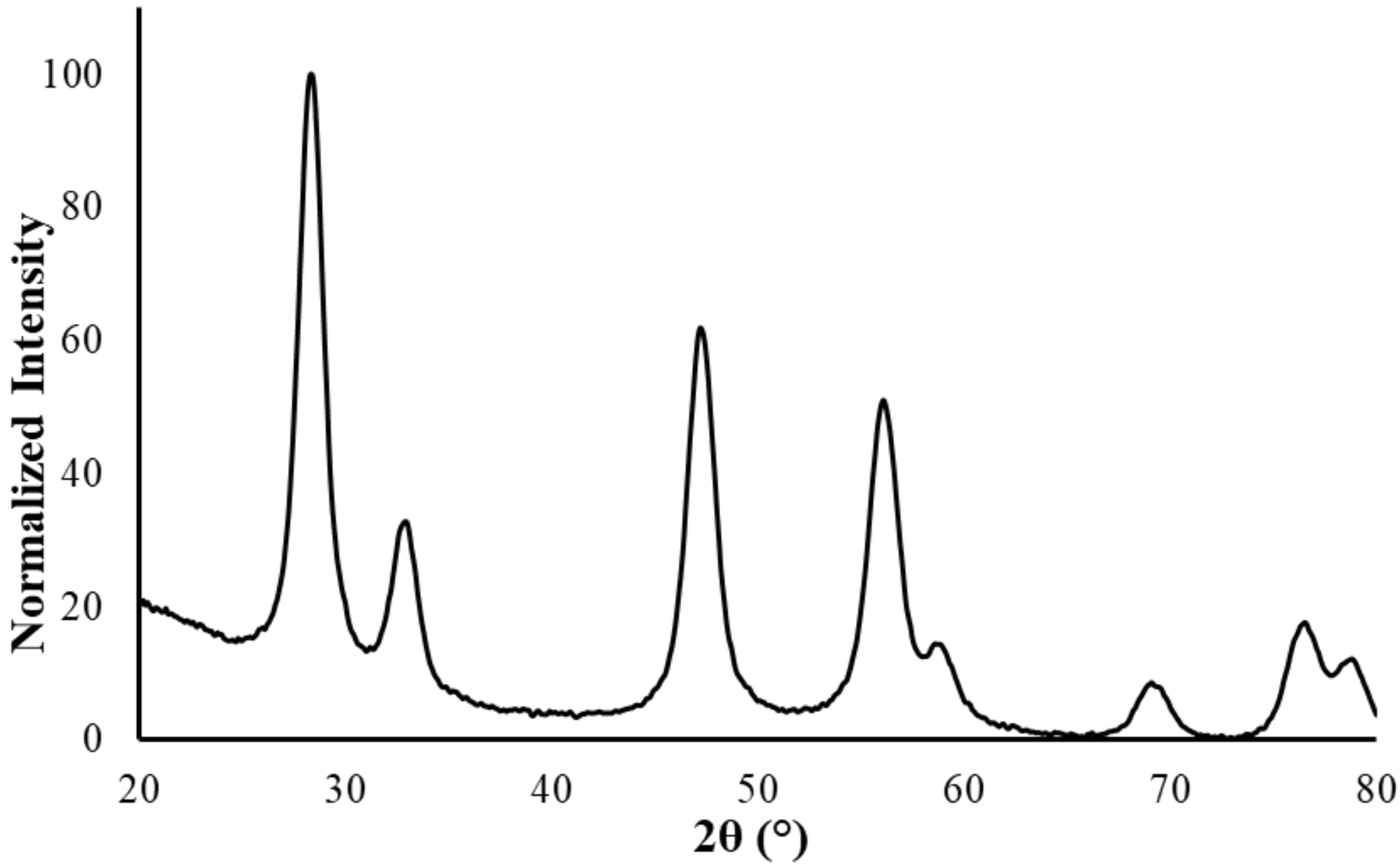

Figure S6: XRD pattern of 2.5%La^{3+}–*n*CeO_2 before the reaction.

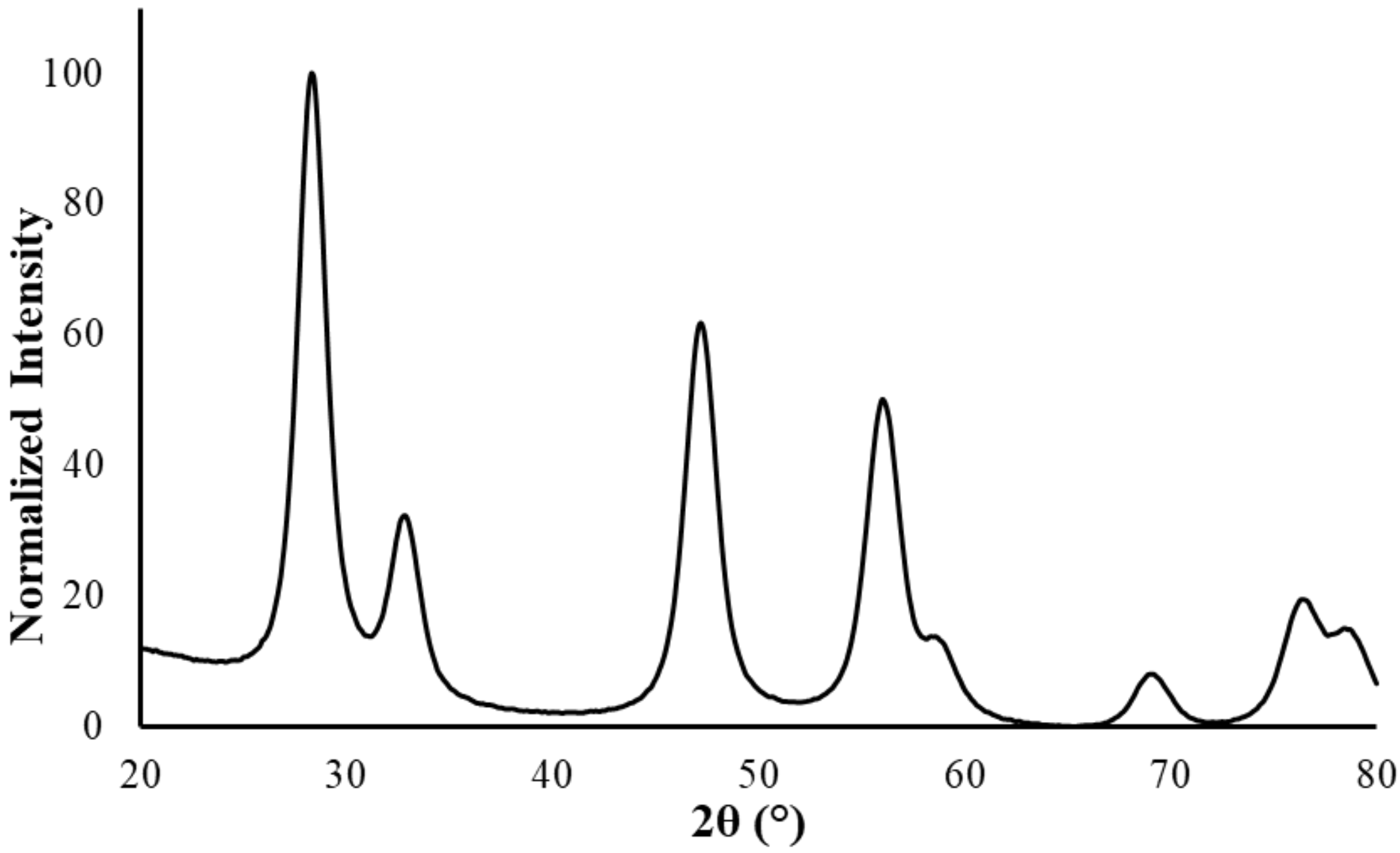

Figure S7: XRD pattern of 5%La^{3+}–*n*CeO_2 before the reaction.

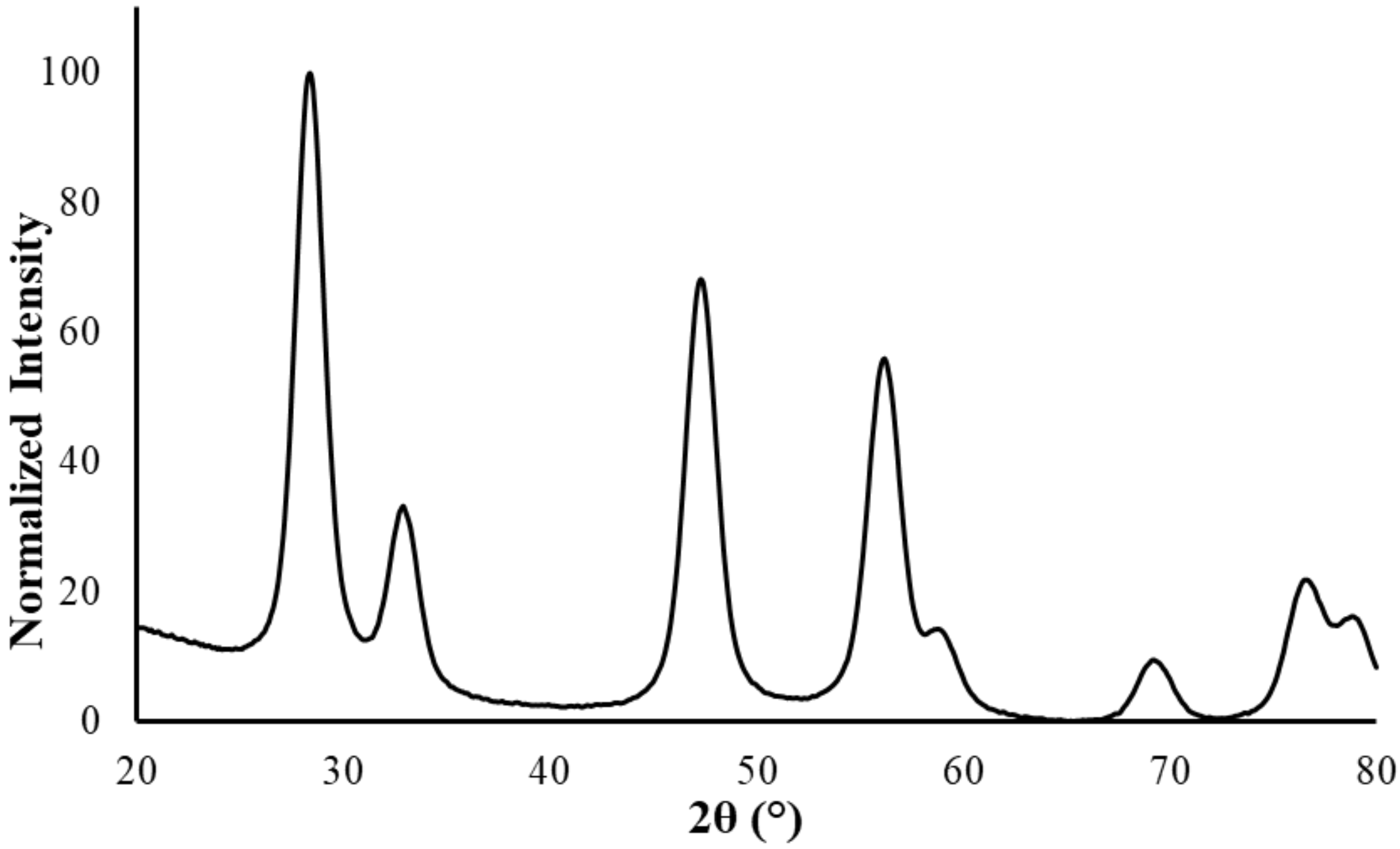

Figure S8: XRD pattern of 2.5%Gd^{3+}–*n*CeO_2 before the reaction.

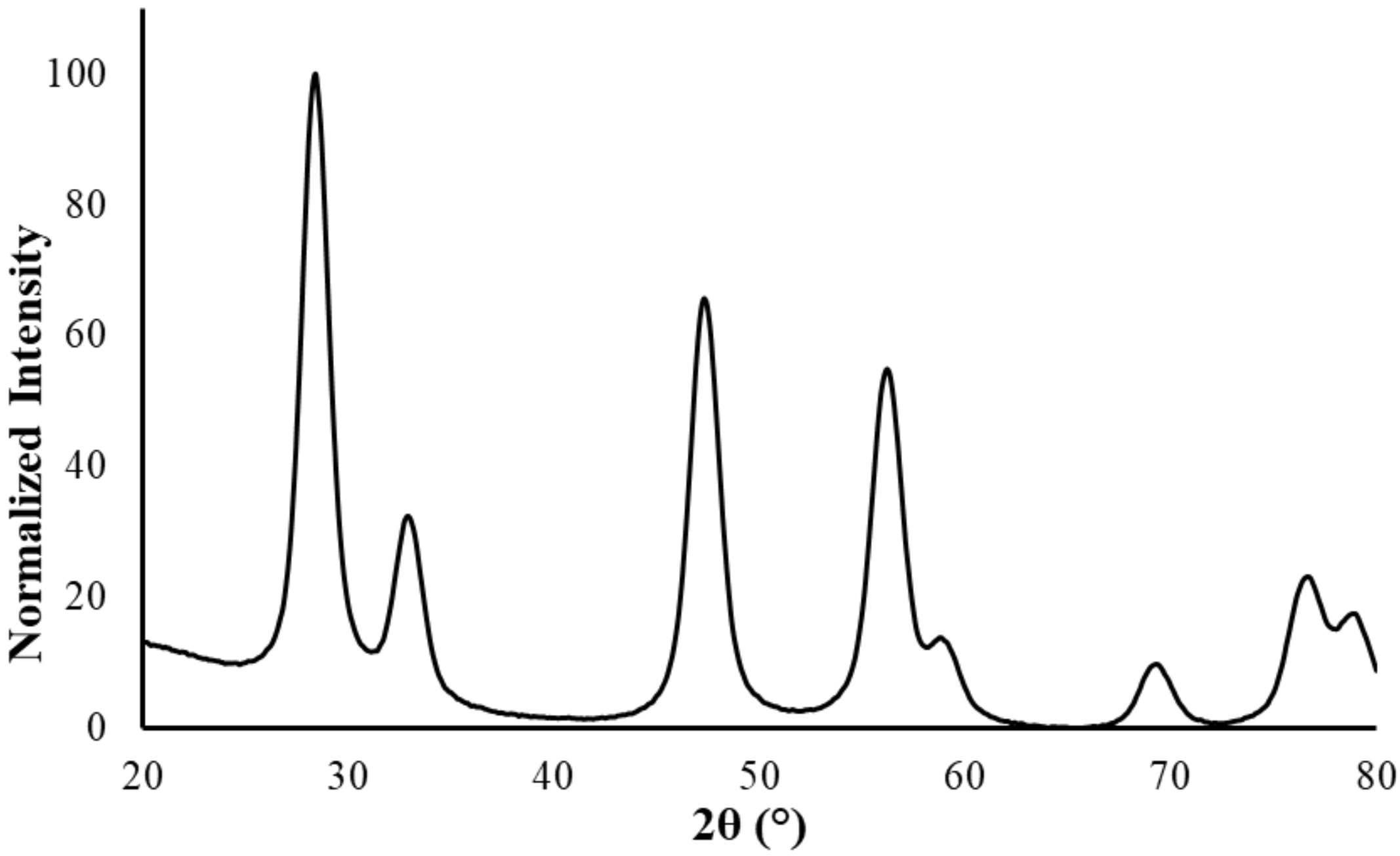

Figure S9: XRD pattern of 5%Gd^{3+}–*n*CeO_2 before the reaction.

The effect of Gd^{3+} and La^{3+} onto CeO_2 structure; full width at half maximum (β), the values of interplanar spacing (d), crystalline size (*D*, Scherrer value), dislocation density (δ value), and number of particles per unit surface area (*N* value) were calculated [2].

According to Table S3 – S5, doping CeO_2 by Gd^{3+} and La^{3+} increases β, which is related to increasing defects in crystal and decreasing the concentration of crystal parts in doped-CeO_2.

And then d value, the lattice spacing was calculated according to equation S3.

$$n\lambda = 2d\sin(\theta) \qquad \text{(S3)}$$

λ is the wavelength of the X-ray (1.5418 Å), θ is the Bragg angle and *n* is the order of diffraction. Tiny changes were recorded in d value between nCeO_2 and doped-CeO_2 (Table S1).

Scherrer equation (Equation S4) shows that dopants decreased the crystal degree of the structure.

$$D = \frac{K\lambda}{\beta \cos(\theta)} \quad \text{(S4)}$$

K is the Scherrer constant, λ is wave length of the X-ray beam (1.5418 Å), β is the Full width at half maximum (FWHM) of the peak and θ is the Bragg angle.

The dislocation density is defined as the length of dislocation lines per unit volume or irregularity in the crystal and is calculatable according to equation S5.

$$\delta = \frac{1}{D^2} \quad \text{(S5)}$$

The number of elements per unit surface area (N value) was calculated by following equation.

$$N = \frac{d}{D^3} \quad \text{(S6)}$$

The XRD measurements and analysis were repeated three times and reported separately in Table S3 – S5.

Table S3: Analysis of XRD patterns related to CeO_2 and doped samples (Step size of 0.1 and speed of 0.5 2θ.sec^{-1}).

Sheet	Sample	2θ (°)	FWHM (radian)	d (nm)	D (nm)	δ (nm^{-2})	N
**111**	*n*CeO_2	28.37	1.483	0.3143	5.5260	0.0327	0.0019
	2.5%La^{3+}–*n*CeO_2	28.33	1.743	0.3148	4.7017	0.0452	0.0030
	5%La^{3+}–*n*CeO_2	28.32	1.898	0.3148	4.3171	0.0537	0.0039
	2.5%Gd^{3+}–*n*CeO_2	28.37	1.840	0.3143	4.4542	0.0504	0.0036
	5%Gd^{3+}–*n*CeO_2	28.39	1.751	0.3141	4.6789	0.0457	0.0031
**200**	*n*CeO_2	32.86	1.576	0.2723	5.2567	0.0362	0.0019
	2.5%La^{3+}–*n*CeO_2	32.77	1.900	0.2730	4.3584	0.0526	0.0033
	5%La^{3+}–*n*CeO_2	32.68	2.200	0.2737	3.7626	0.0706	0.0051
	2.5%Gd^{3+}–*n*CeO_2	32.82	1.926	0.2727	4.2995	0.0541	0.0034
	5%Gd^{3+}–*n*CeO_2	32.84	1.796	0.2725	4.6109	0.0470	0.0028
**220**	*n*CeO_2	47.30	1.441	0.1920	6.0193	0.0276	0.0009
	2.5%La^{3+}–*n*CeO_2	47.25	1.588	0.1922	5.4619	0.0335	0.0012
	5%La^{3+}–*n*CeO_2	47.20	1.816	0.1924	4.7740	0.0439	0.0018
	2.5%Gd^{3+}–*n*CeO_2	47.31	1.738	0.1920	4.9899	0.0402	0.0015
	5%Gd^{3+}–*n*CeO_2	47.32	1.640	0.1919	5.2880	0.0358	0.0013
**311**	*n*CeO_2	56.18	1.518	0.1636	5.9332	0.0284	0.0008
	2.5%La^{3+}–*n*CeO_2	56.12	1.728	0.1637	5.2111	0.0368	0.0012
	5%La^{3+}–*n*CeO_2	56.08	2.029	0.1638	4.4354	0.0508	0.0019
	2.5%Gd^{3+}–*n*CeO_2	56.20	1.897	0.1635	4.7473	0.0444	0.0015
	5%Gd^{3+}–*n*CeO_2	56.21	1.782	0.1635	5.0544	0.0391	0.0013

Table S4: Analysis of XRD patterns related to CeO_2 and doped samples (Step size of 0.1 and speed of 0.5 2θ.sec^{-1}).

**Sheet**	**Sample**	**2θ (°)**	**FWHM (radian)**	**d (nm)**	**D (nm)**	**δ (nm^{-2})**	**N**
**111**	nCeO$_2$	28.39	1.264	0.3141	6.483	0.0238	0.0012
	2.5%La^{3+}–nCeO$_2$	28.33	1.423	0.3147	5.759	0.0301	0.0016
	5%La^{3+}–nCeO$_2$	28.32	1.592	0.3149	5.145	0.0378	0.0023
	2.5%Gd^{3+}–nCeO$_2$	28.37	1.570	0.3143	5.219	0.0367	0.0022
	5%Gd^{3+}–nCeO$_2$	28.39	1.511	0.3141	5.424	0.0340	0.0020
**200**	nCeO$_2$	32.95	1.097	0.2717	7.553	0.0175	0.0006
	2.5%La^{3+}–nCeO$_2$	32.88	1.263	0.2722	6.560	0.0232	0.0010
	5%La^{3+}–nCeO$_2$	32.85	1.362	0.2724	6.079	0.0270	0.0012
	2.5%Gd^{3+}–nCeO$_2$	32.95	1.357	0.2717	6.106	0.0268	0.0012
	5%Gd^{3+}–nCeO$_2$	32.95	1.298	0.2716	6.382	0.0246	0.0010
**220**	nCeO$_2$	47.33	1.395	0.1919	6.218	0.0259	0.0008
	2.5%La^{3+}–nCeO$_2$	47.25	1.574	0.1922	5.507	0.0330	0.0012
	5%La^{3+}–nCeO$_2$	47.20	1.785	0.1924	4.858	0.0424	0.0017
	2.5%Gd^{3+}–nCeO$_2$	47.30	1.726	9.1920	5.026	0.0396	0.0015
	5%Gd^{3+}–nCeO$_2$	47.32	1.656	0.1919	5.237	0.0365	0.0013
**311**	nCeO$_2$	56.21	1.477	0.1635	6.098	0.0270	0.0007
	2.5%La^{3+}–nCeO$_2$	56.13	1.730	0.1637	5.203	0.0370	0.0012
	5%La^{3+}–nCeO$_2$	56.10	1.998	0.1638	4.506	0.0492	0.0018
	2.5%Gd^{3+}–nCeO$_2$	56.22	1.908	0.1635	4.722	0.0445	0.0015
	5%Gd^{3+}–nCeO$_2$	56.22	1.802	0.1635	4.998	0.0400	0.0013

Table S5: Analysis of XRD patterns related to CeO_2 and doped samples (Step size of 0.02 and speed of 1 2θ.sec^{-1}).

Sheet	Sample	2θ (°)	FWHM (radian)	d (nm)	D (nm)	δ (nm^{-2})	N
**111**	*n*CeO_2	28.33	1.166	0.3147	7.027	0.0202	0.0009
	2.5%La^{3+}–*n*CeO_2	28.35	2.990	0.3146	2.740	0.1332	0.0153
	5%La^{3+}–*n*CeO_2	28.28	3.166	0.3153	2.587	0.1493	0.0182
	2.5%Gd^{3+}–*n*CeO_2	28.33	2.624	0.3148	3.123	0.1025	0.0103
	5%Gd^{3+}–*n*CeO_2	28.40	2.257	0.3140	3.631	0.0759	0.0066
**200**	*n*CeO_2	32.89	1.007	0.2721	8.226	0.0148	0.0005
	2.5%La^{3+}–*n*CeO_2	32.46	3.250	0.2756	2.545	0.1543	0.0167
	5%La^{3+}–*n*CeO_2	32.50	3.397	0.2753	2.436	0.1685	0.0190
	2.5%Gd^{3+}–*n*CeO_2	32.49	3.167	0.2753	2.613	0.1465	0.0154
	5%Gd^{3+}–*n*CeO_2	32.61	2.846	0.2744	2.908	0.1183	0.0112
**220**	*n*CeO_2	47.27	1.291	0.1921	6.716	0.0221	0.0006
	2.5%La^{3+}–*n*CeO_2	47.20	3.344	0.1924	2.592	0.1488	0.0110
	5%La^{3+}–*n*CeO_2	47.18	3.360	0.1925	2.580	0.1502	0.0112
	2.5%Gd^{3+}–*n*CeO_2	47.19	2.841	0.1924	3.052	0.1074	0.0068
	5%Gd^{3+}–*n*CeO_2	47.27	2.330	0.1921	3.722	0.0722	0.0037
**311**	*n*CeO_2	56.16	1.541	0.1637	5.843	0.0293	0.0008
	2.5%La^{3+}–*n*CeO_2	56.28	4.132	0.1633	2.180	0.2104	0.0158
	5%La^{3+}–*n*CeO_2	56.36	4.482	0.1631	2.011	0.2473	0.0200
	2.5%Gd^{3+}–*n*CeO_2	56.39	3.964	0.1630	2.273	0.1935	0.0139
	5%Gd^{3+}–*n*CeO_2	56.25	2.876	0.1634	3.132	0.1019	0.0053

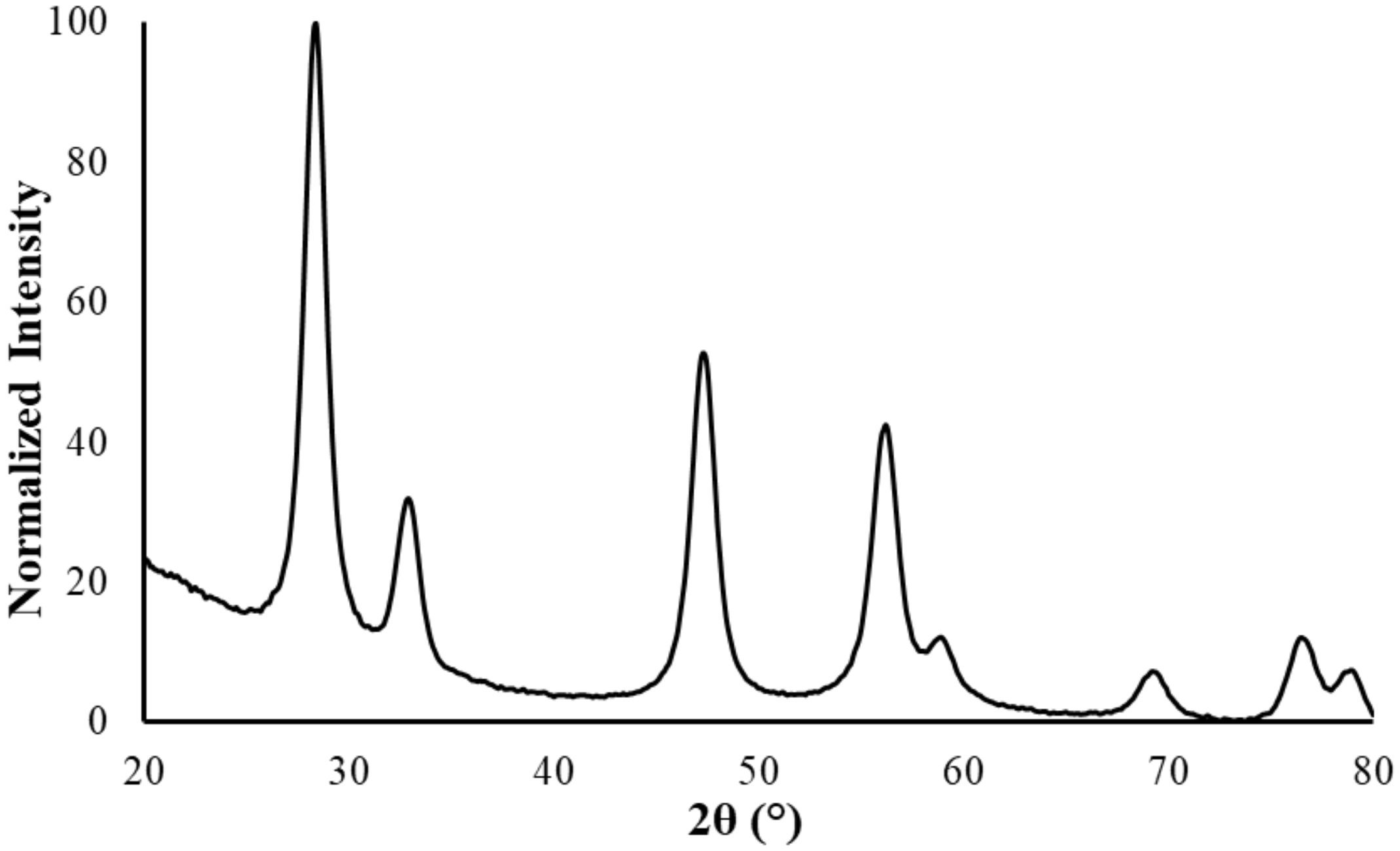

Figure S10: XRD pattern of *n*CeO_2 after the reaction.

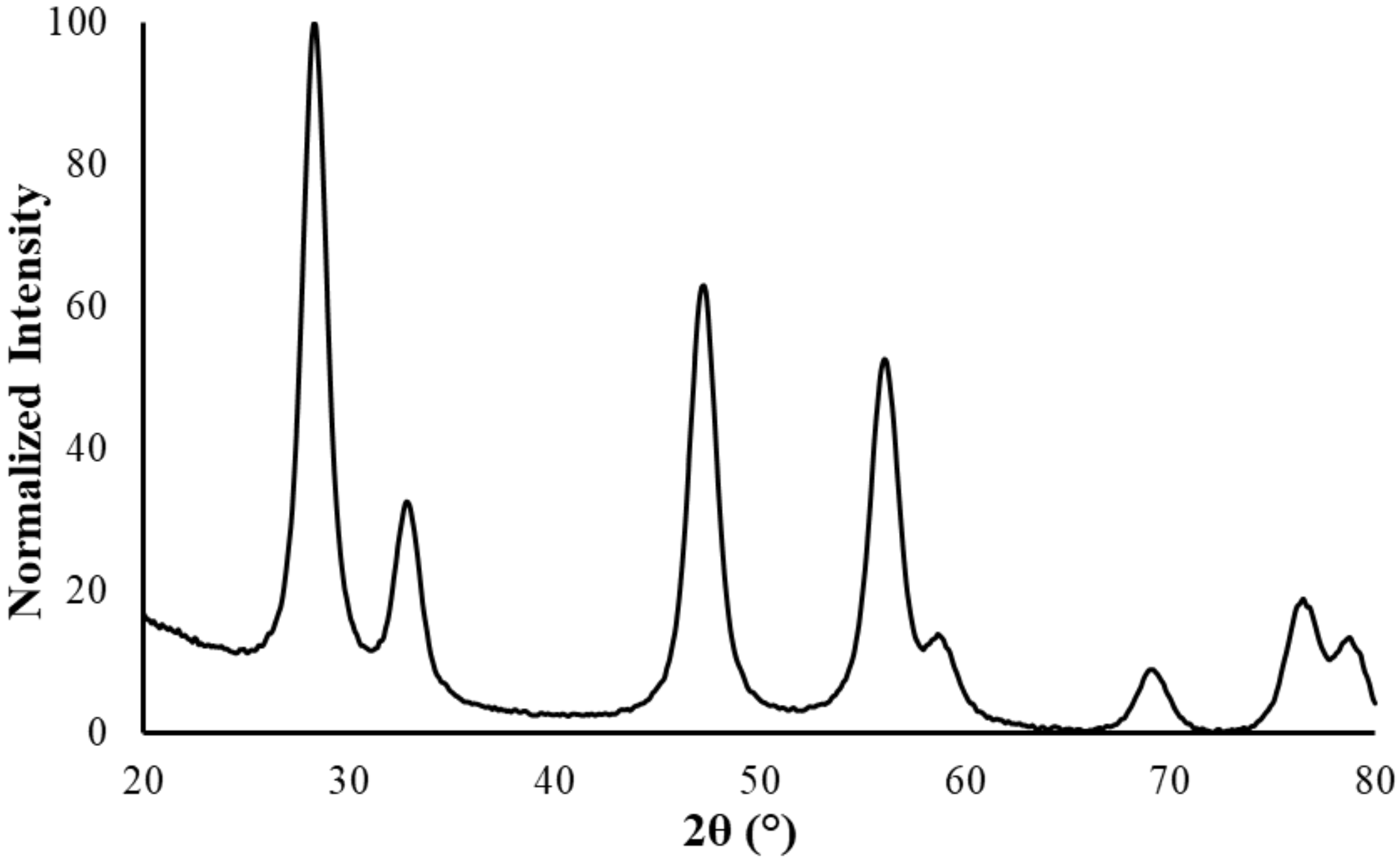

Figure S11: XRD pattern of 2.5%La^{3+}–*n*CeO_2 after the reaction.

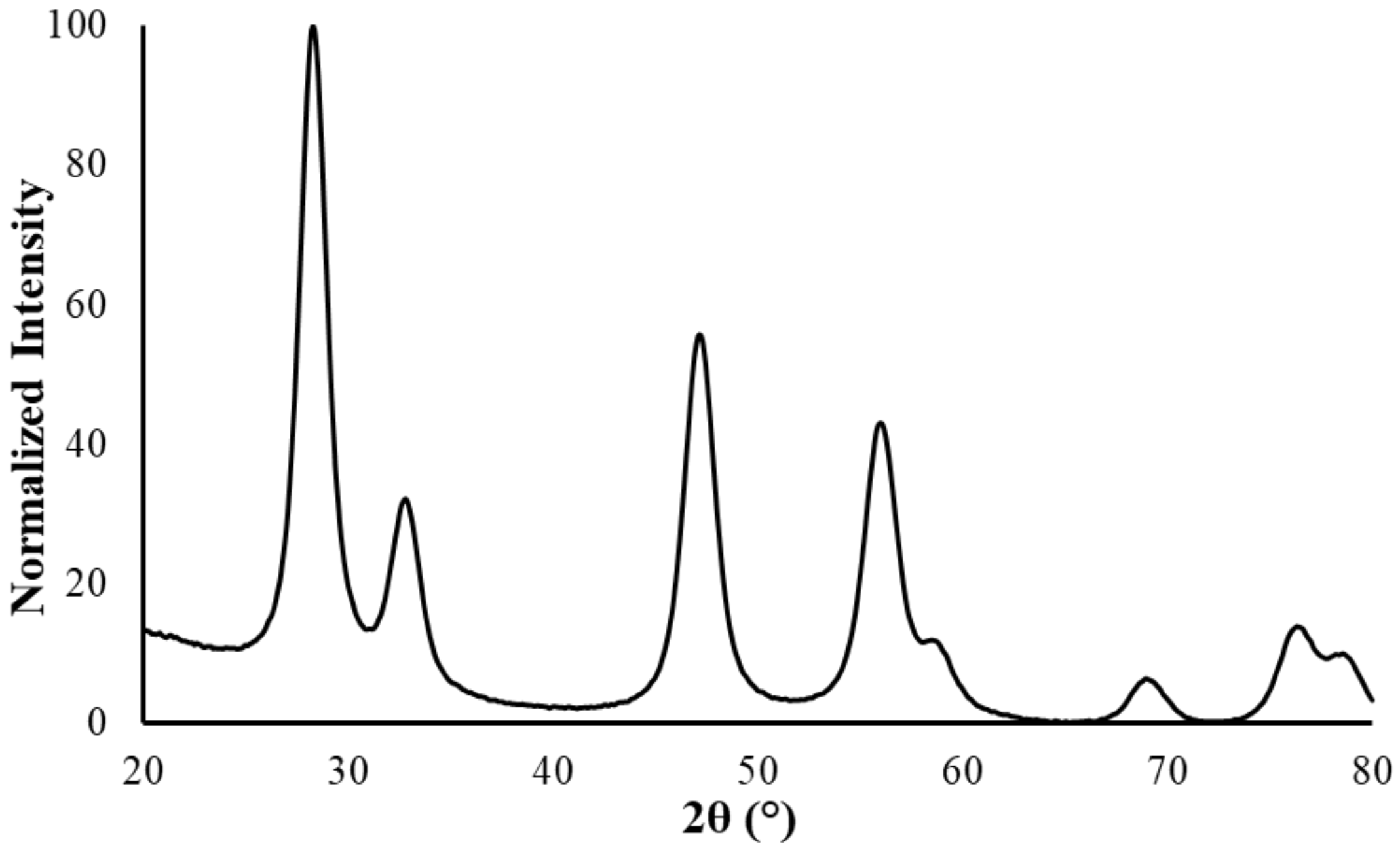

Figure S12: XRD pattern of 5%La^{3+}–*n*CeO_2 after the reaction.

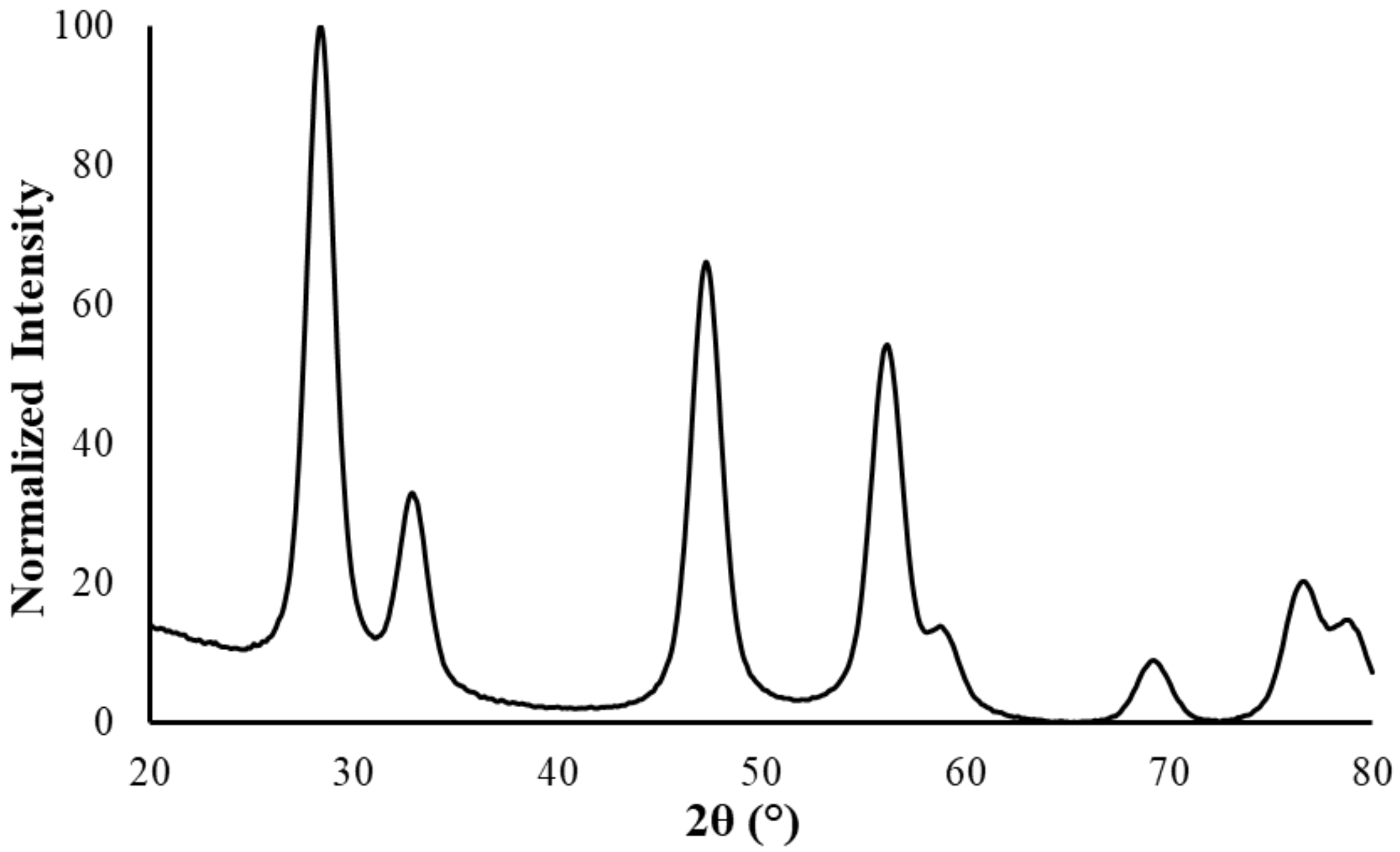

Figure S13: XRD pattern of 2.5%Gd^{3+}–*n*CeO_2 after the reaction.

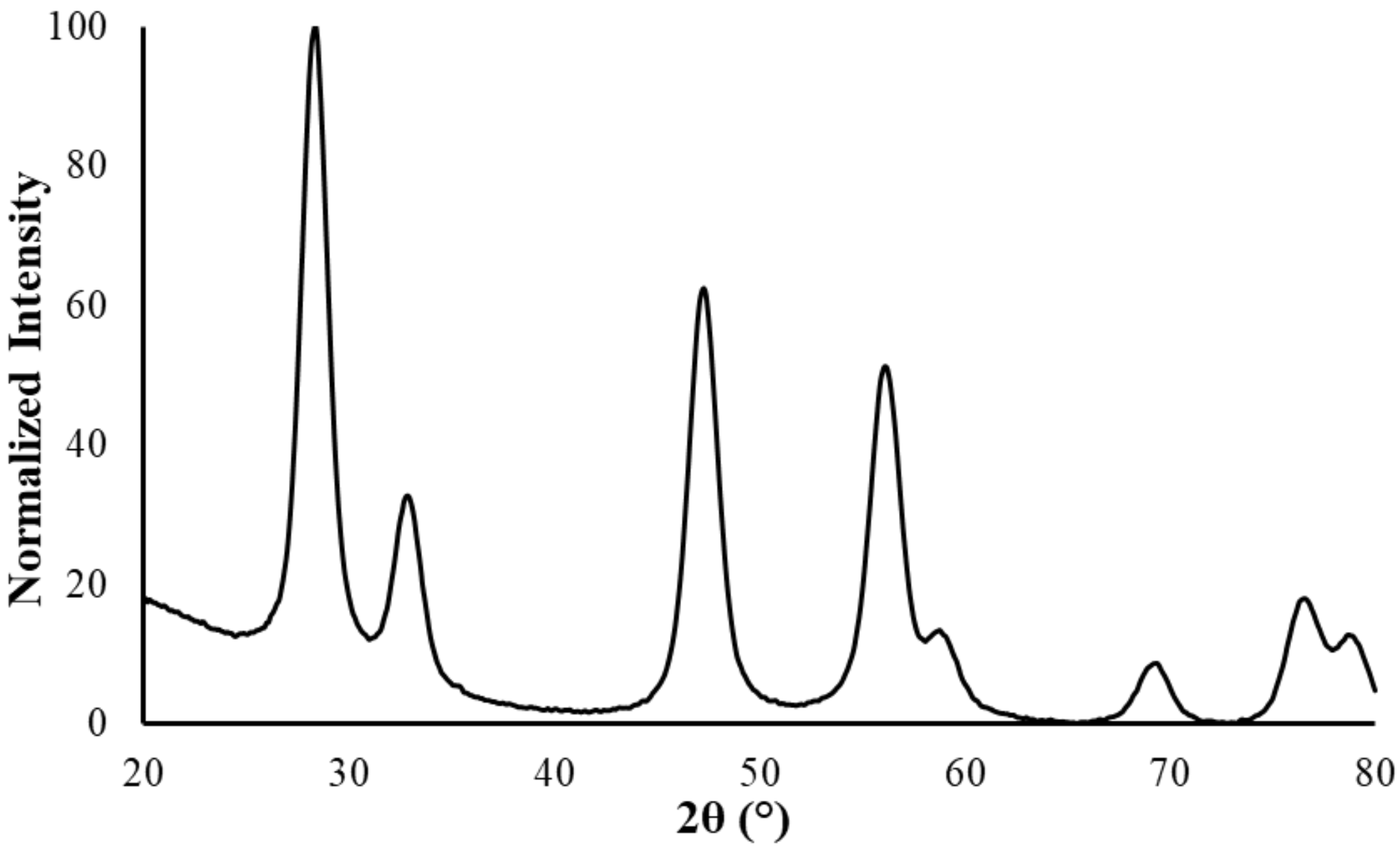

Figure S14: XRD pattern of 5%Gd^{3+}–*n*CeO_2 after the reaction.

Figure S15-S24 are related to the Raman spectra, before and after the reaction.

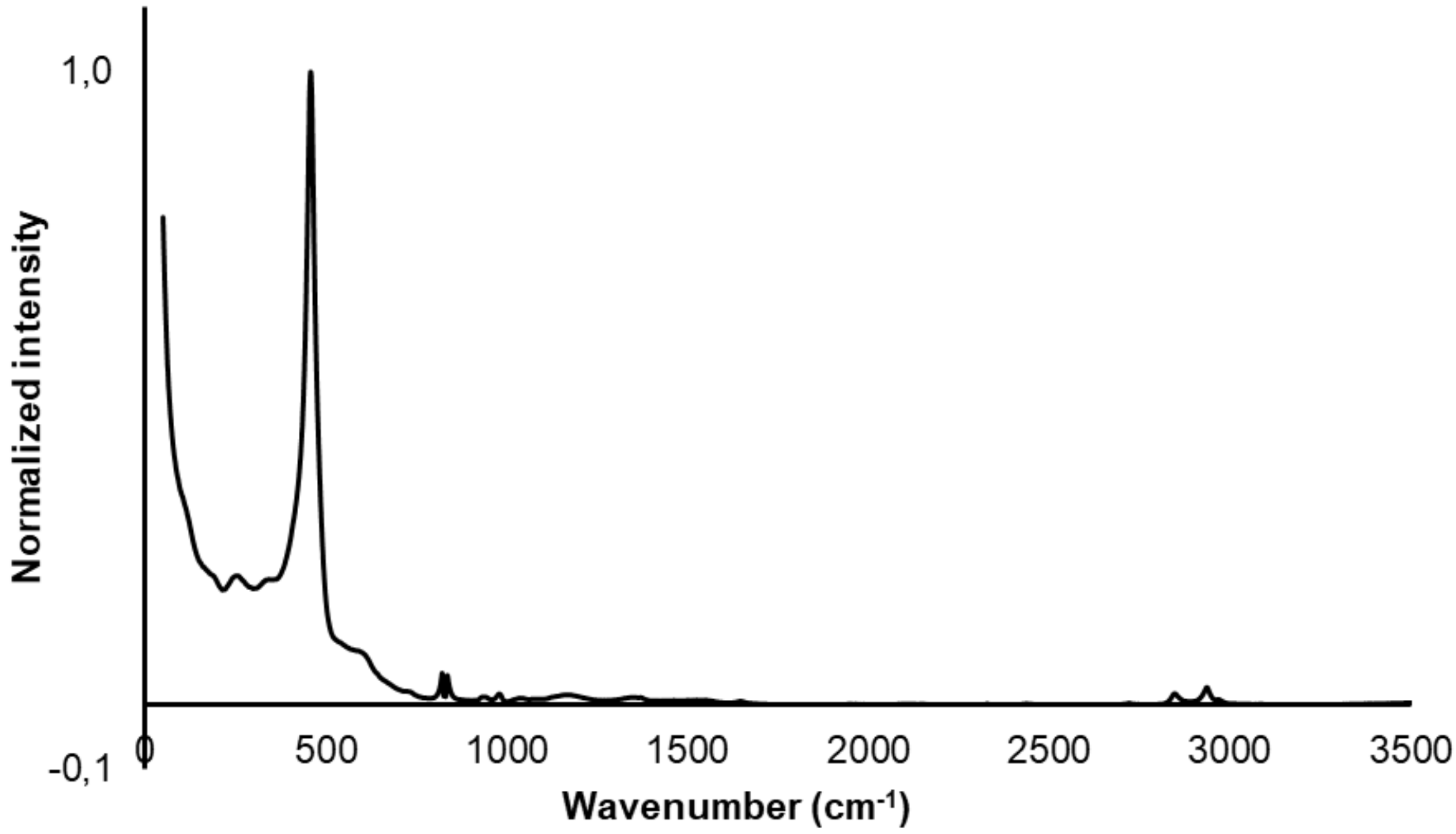

Figure S15: Raman spectra of nCeO$_2$ before the reaction.

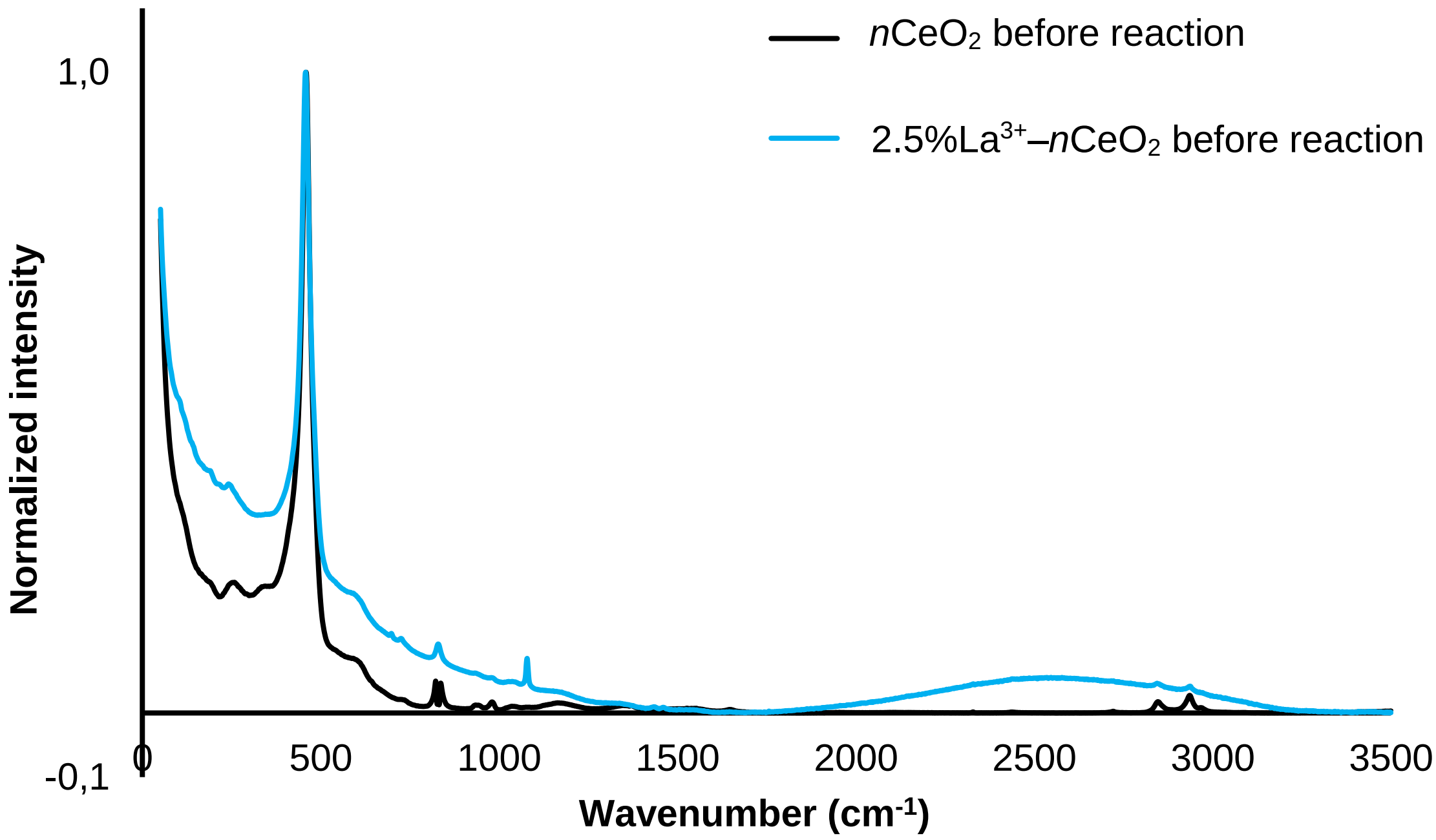

Figure S16: Raman spectra of 2.5%La^{3+}–nCeO$_2$ before the reaction.

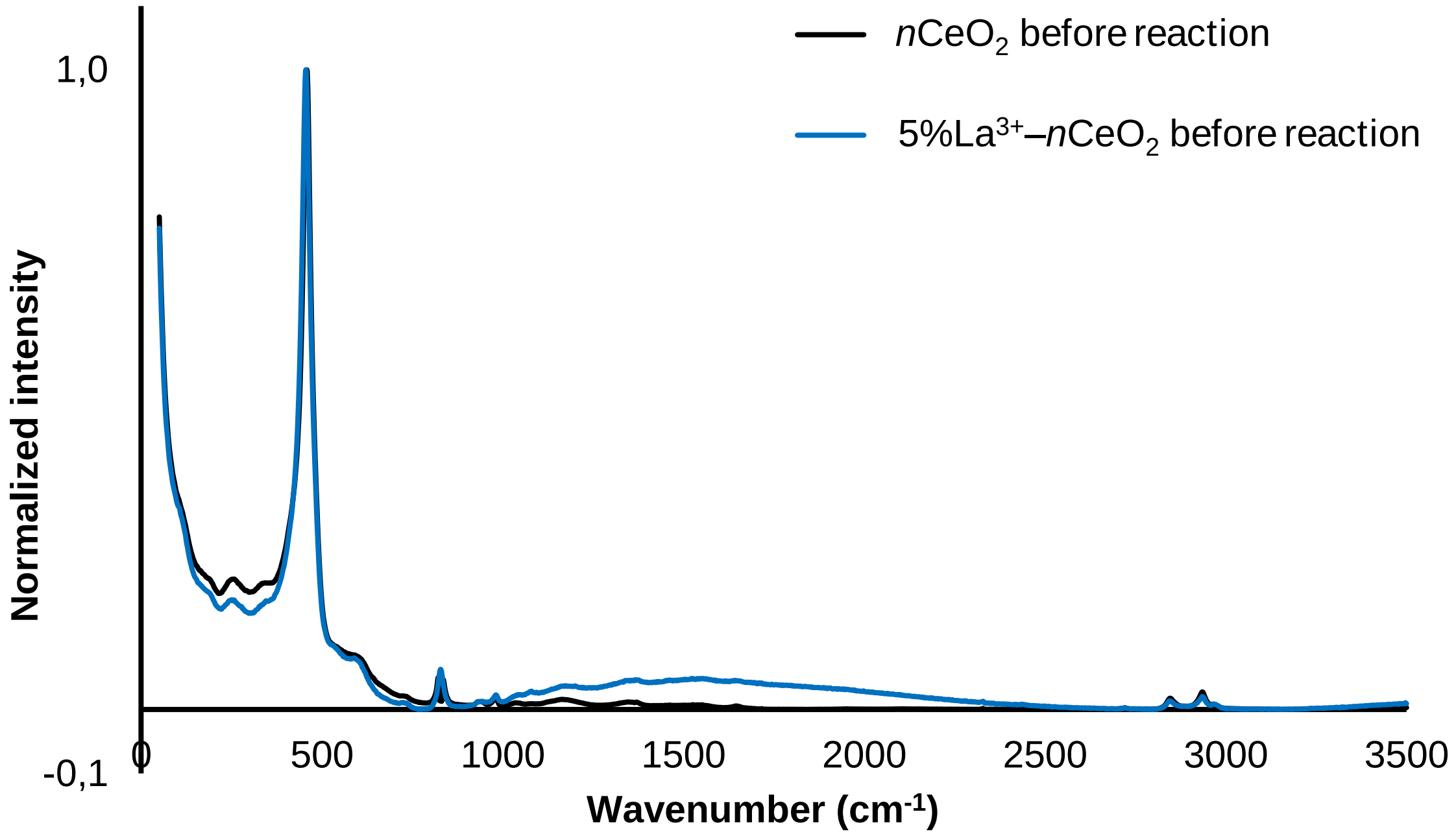

Figure S17: Raman spectra of 5%La^{3+}–*n*CeO_2 before the reaction.

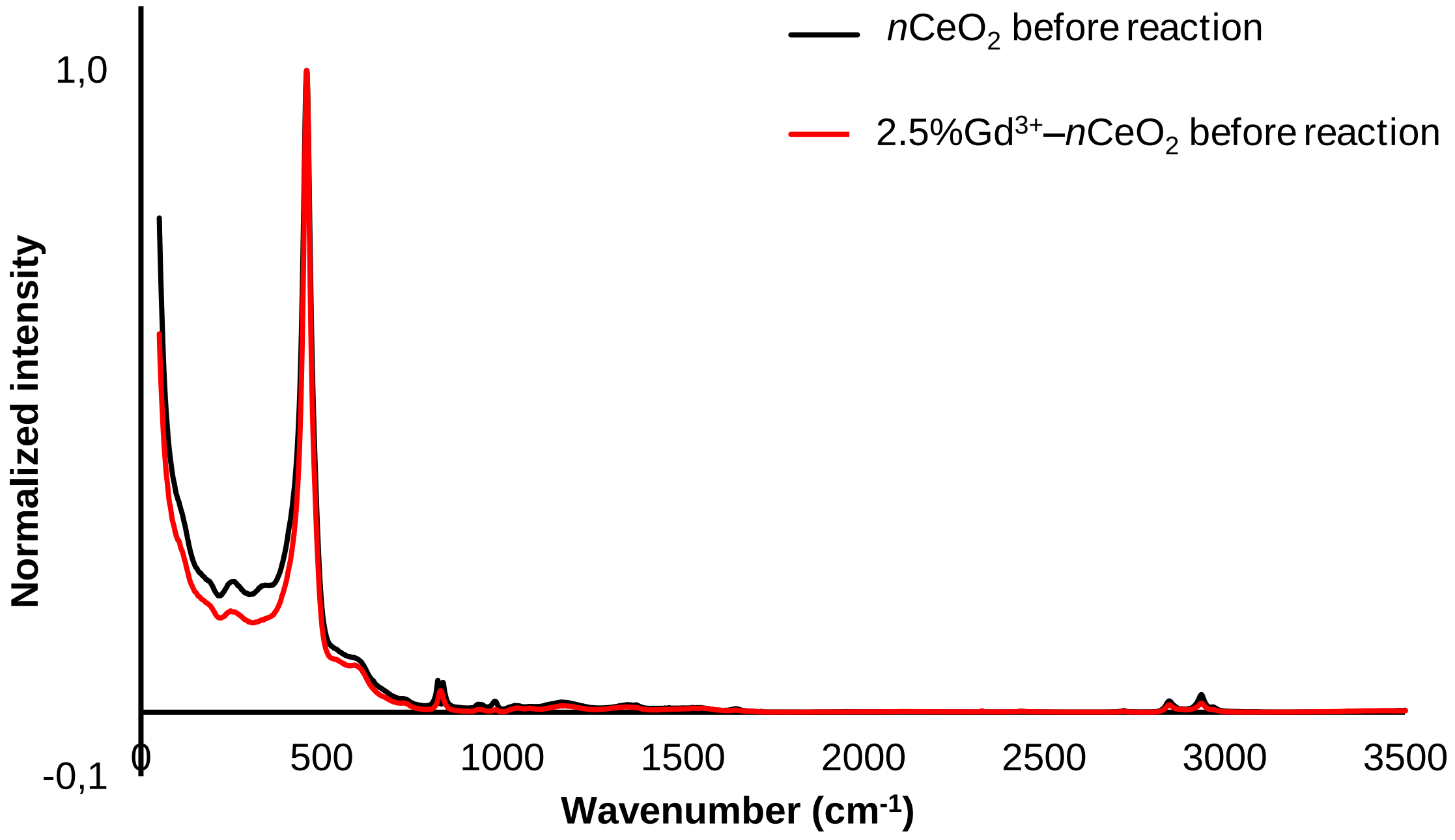

Figure S18: Raman spectra of 2.5%Gd^{3+}–*n*CeO_2 before the reaction.

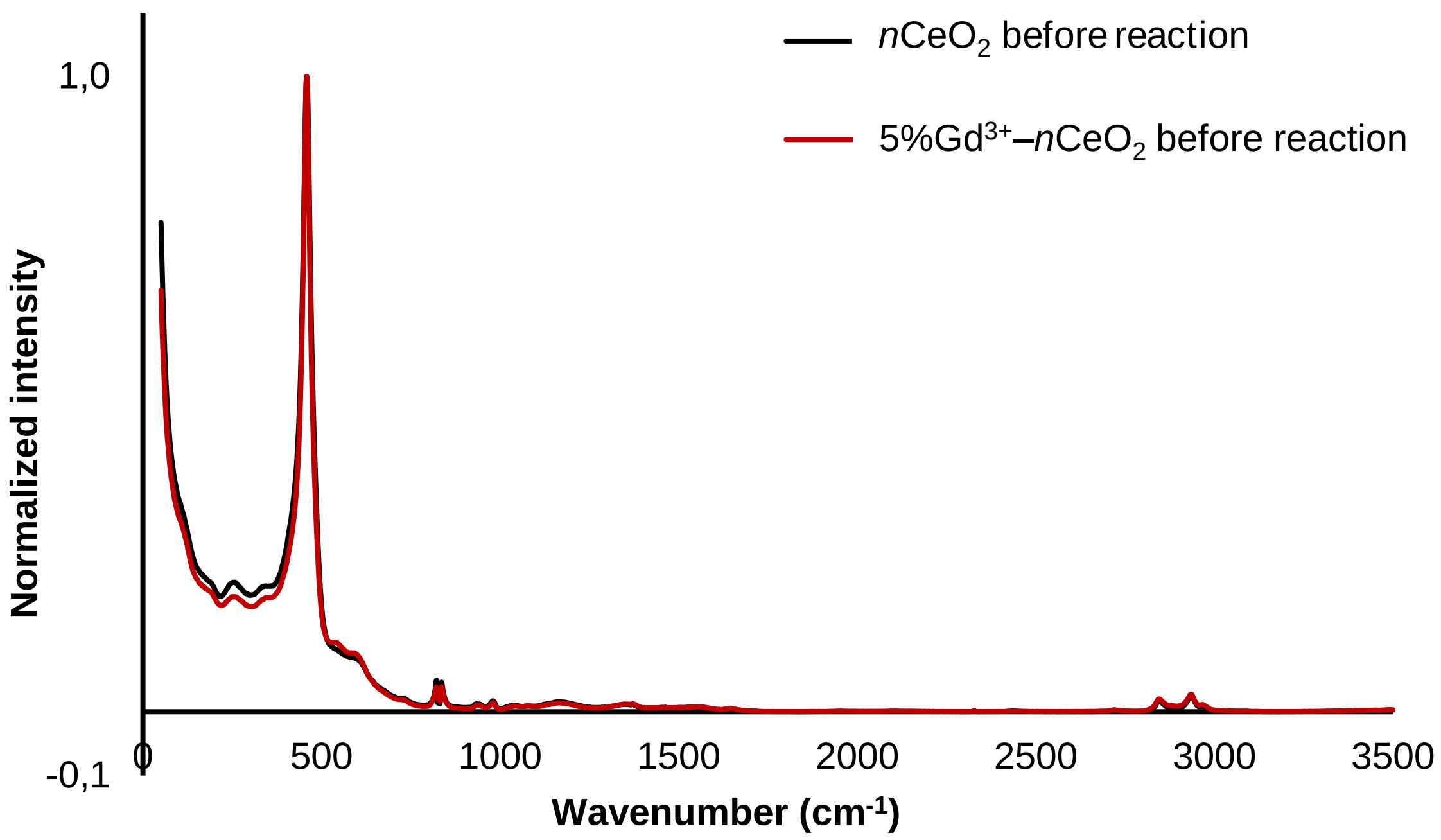

Figure S19: Raman spectra of 5%Gd^{3+}–*n*CeO_2 before the reaction.

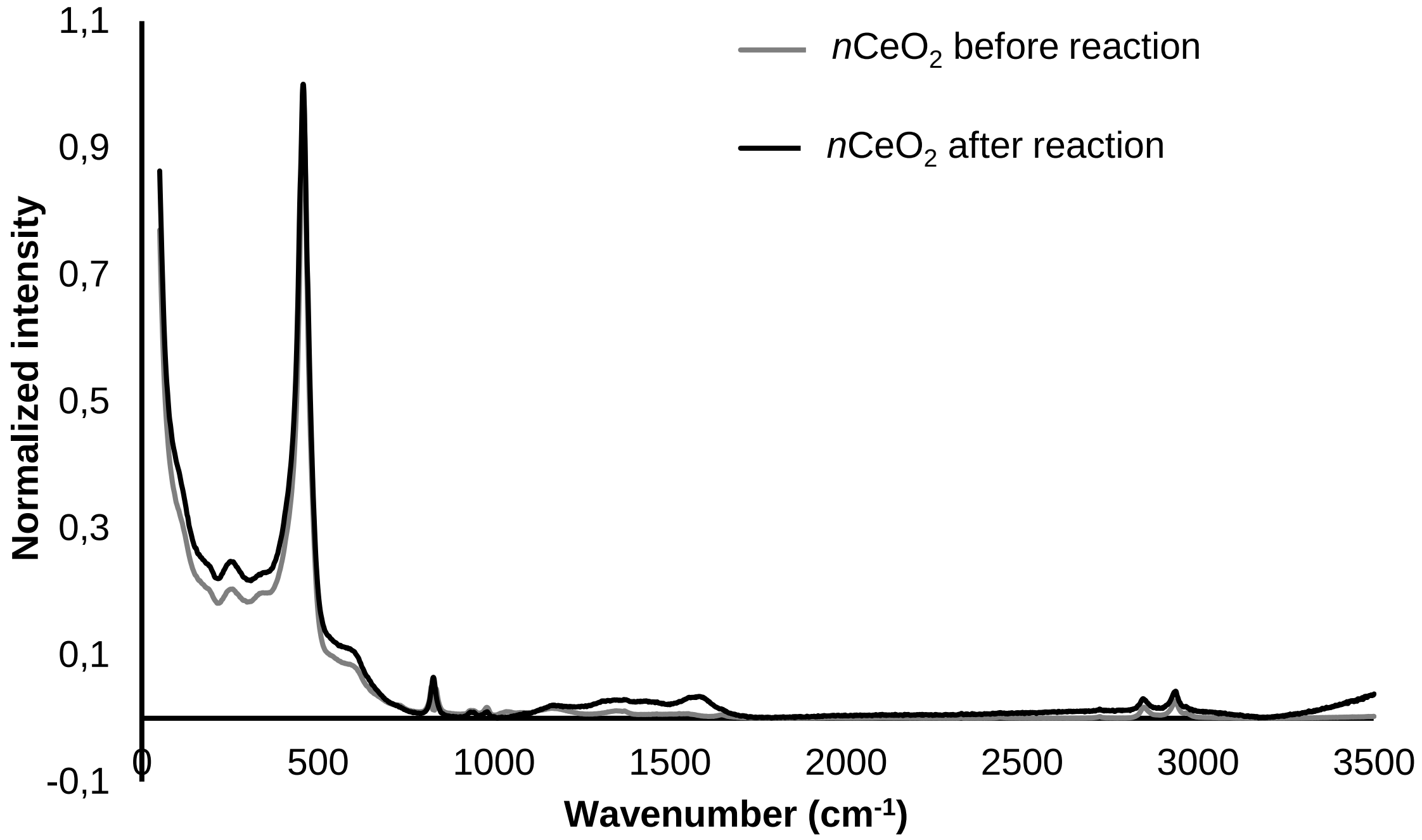

Figure S20: Raman spectra of *n*CeO_2 after the reaction.

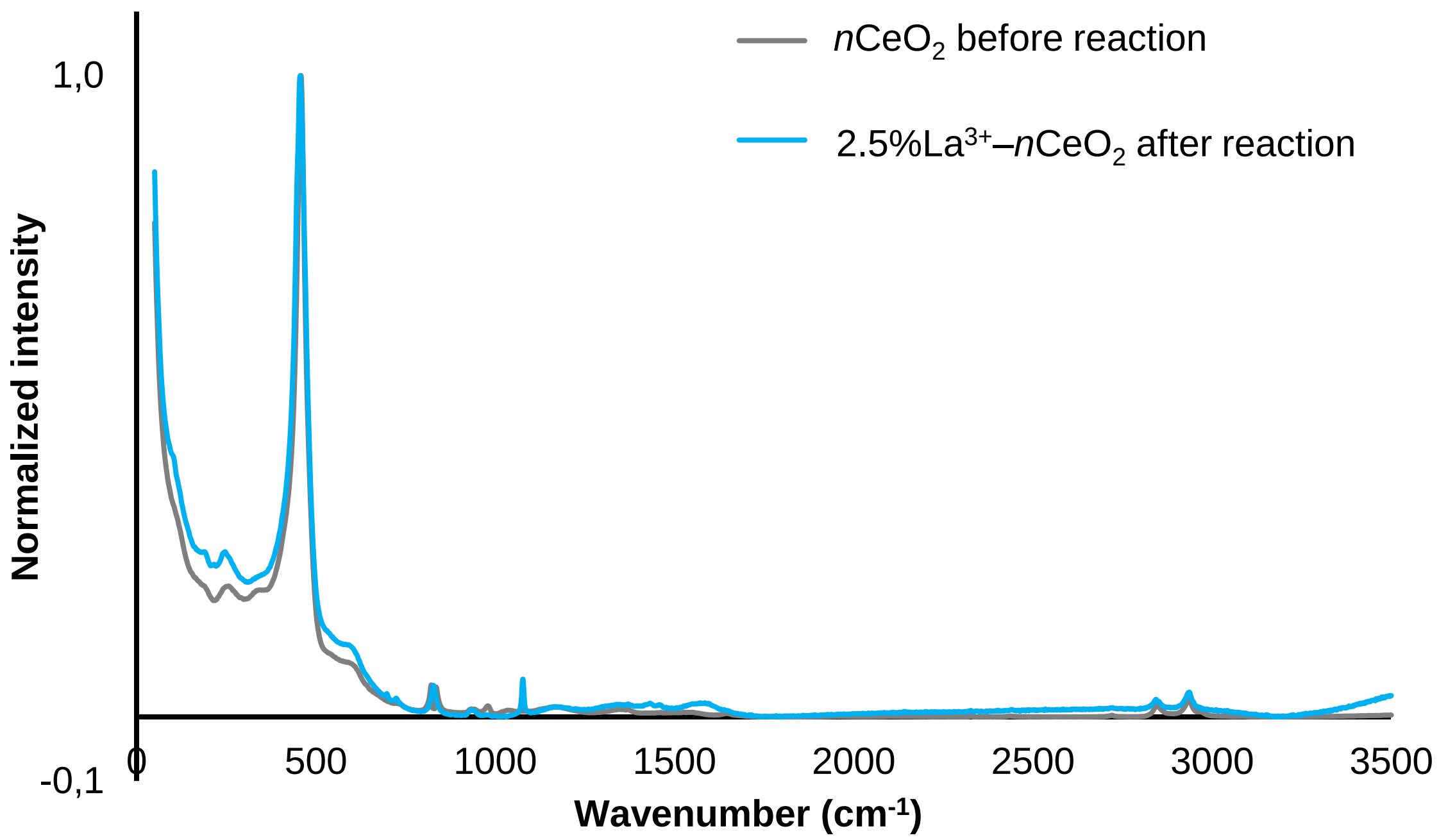

Figure S21: Raman spectra of 2.5%La^{3+}–*n*CeO_2 after the reaction.

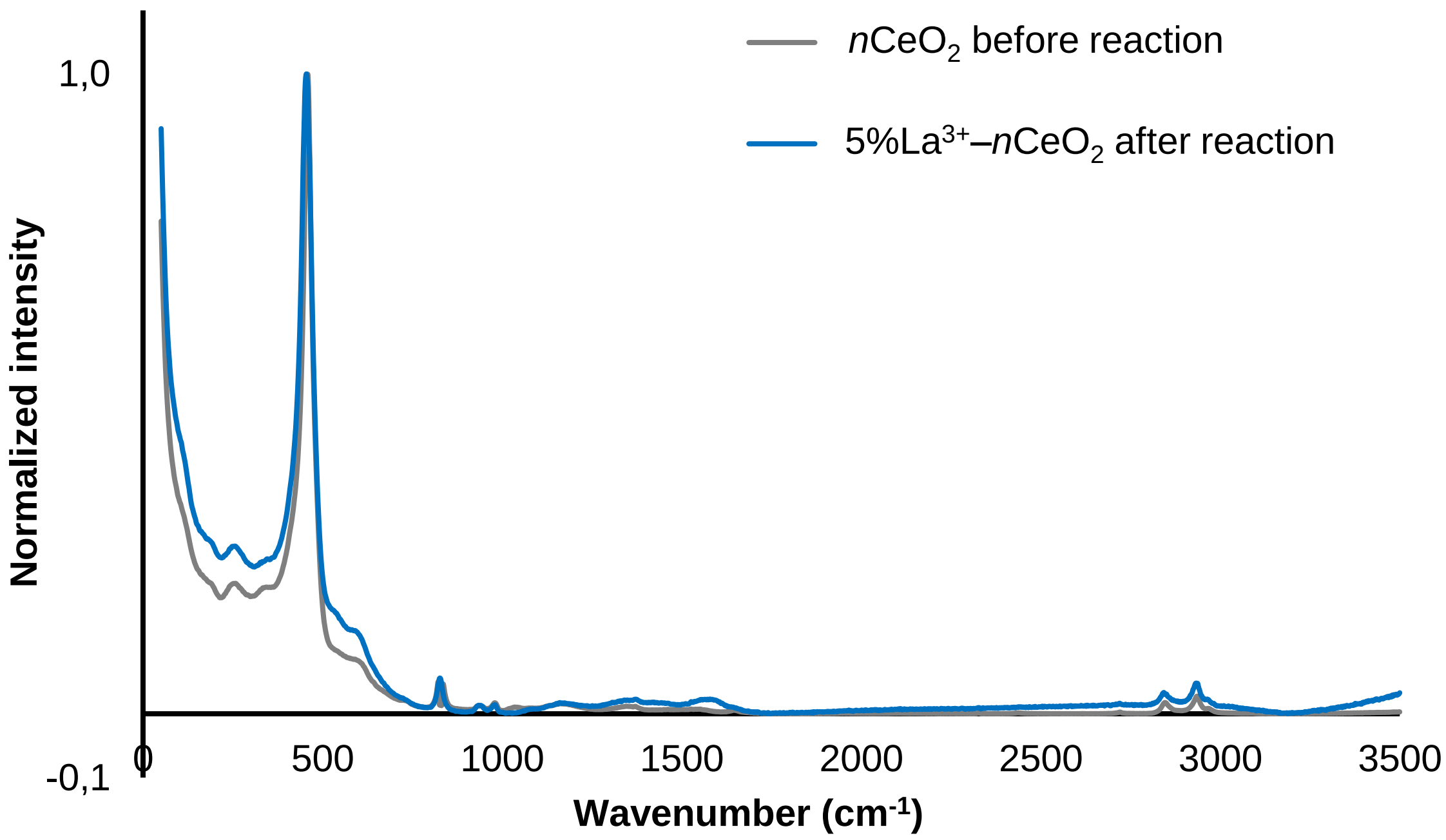

Figure S22: Raman spectra of 5%La^{3+}–*n*CeO_2 after the reaction.

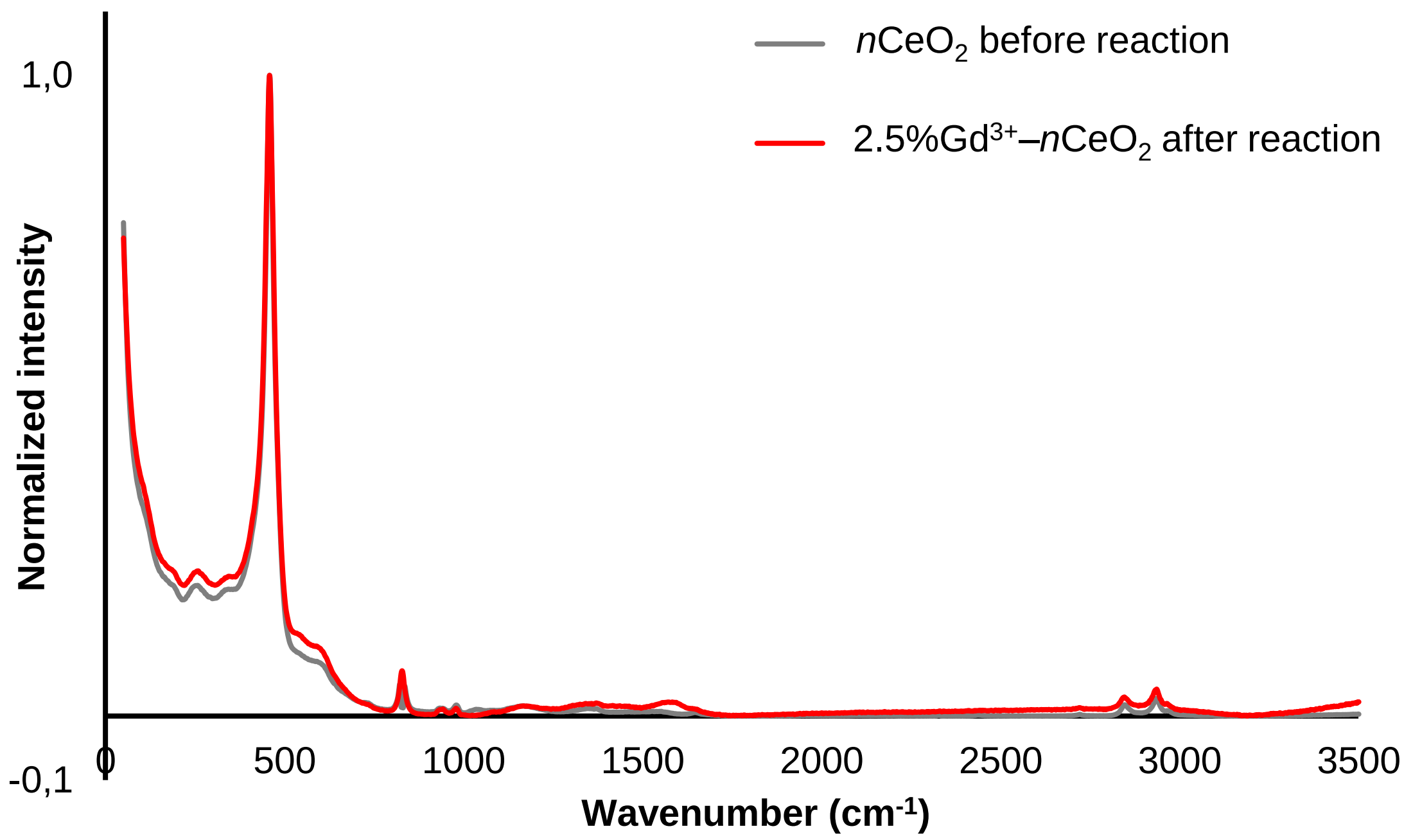

Figure S23: Raman spectra of 2.5%Gd^{3+}–*n*CeO_2 after the reaction.

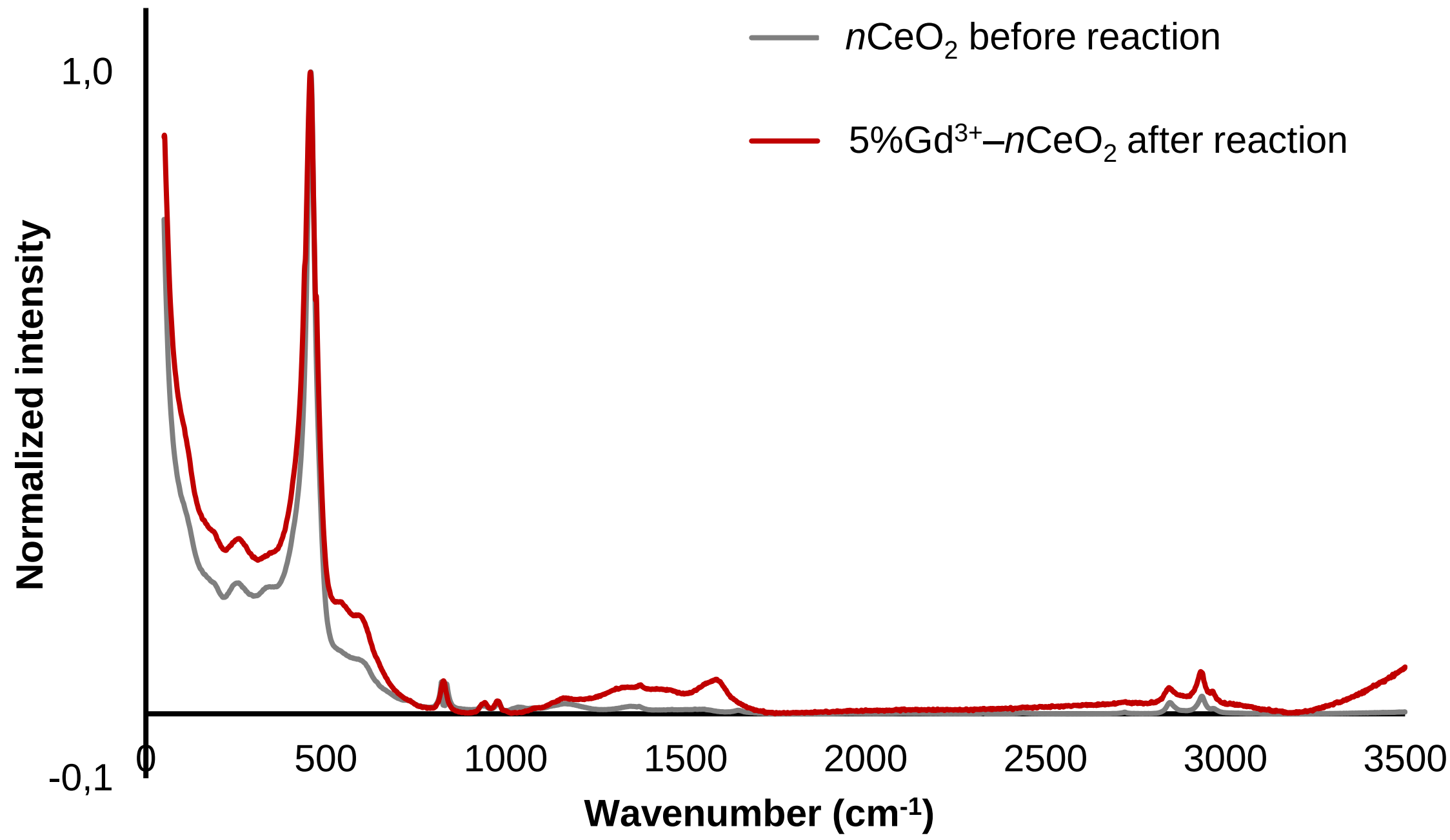

Figure S24: Raman spectra of 5%Gd^{3+}–*n*CeO_2 after the reaction.

Figure S25 shows the scanning electron microscopy (SEM) images of the samples.

a
b
c
d
e

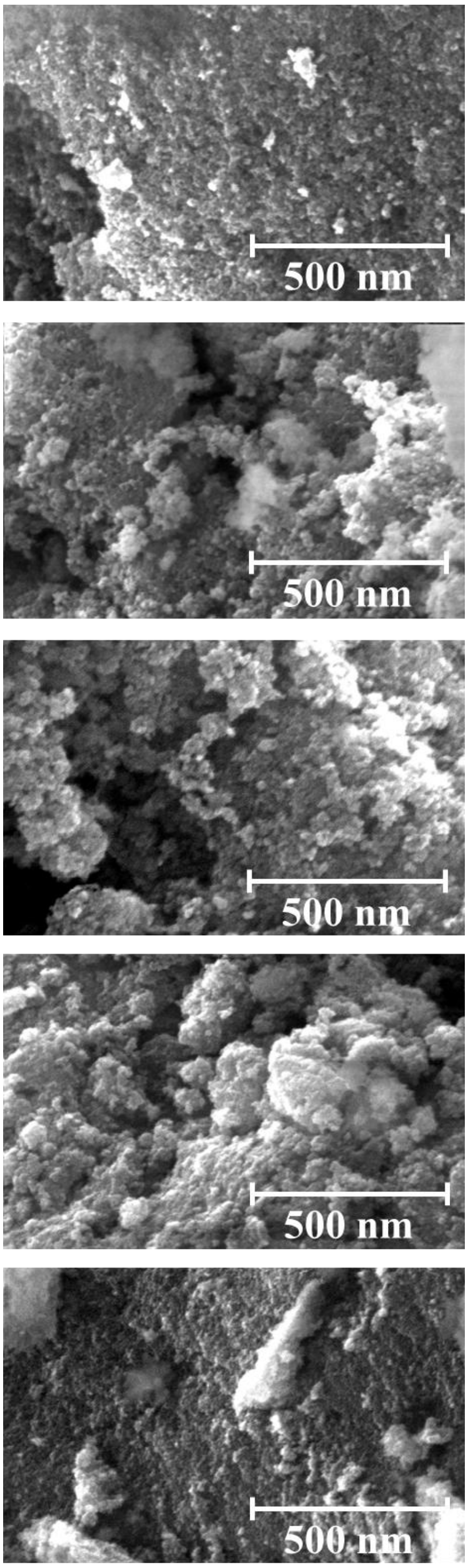

Figure S25: SEM images of (a) *n*CeO_2 (b) 2.5%La^{3+}–*n*CeO_2, (c) 5%La^{3+}–*n*CeO_2, (d) 2.5%Gd^{3+}–*n*CeO_2 and (e) 5%Gd^{3+}–*n*CeO_2.

The BET surface area of each catalyst is written in Table S6.

Table S6: The summary of BET analysis of the samples.

**Sample**	**Surface Area ($m^2.g^{-1}$)**
nCeO_2	134.600
2.5%La^{3+}–*n*CeO_2	134.065
5%La^{3+}–*n*CeO_2	137.837
2.5%Gd^{3+}–*n*CeO_2	148.047
5%Gd^{3+}–*n*CeO_2	138.110

Results of I_{CO}

Figure S26 and S27 plot I_{CO} versus passing time of reaction under the condition of free potential and applied potential, respectively.

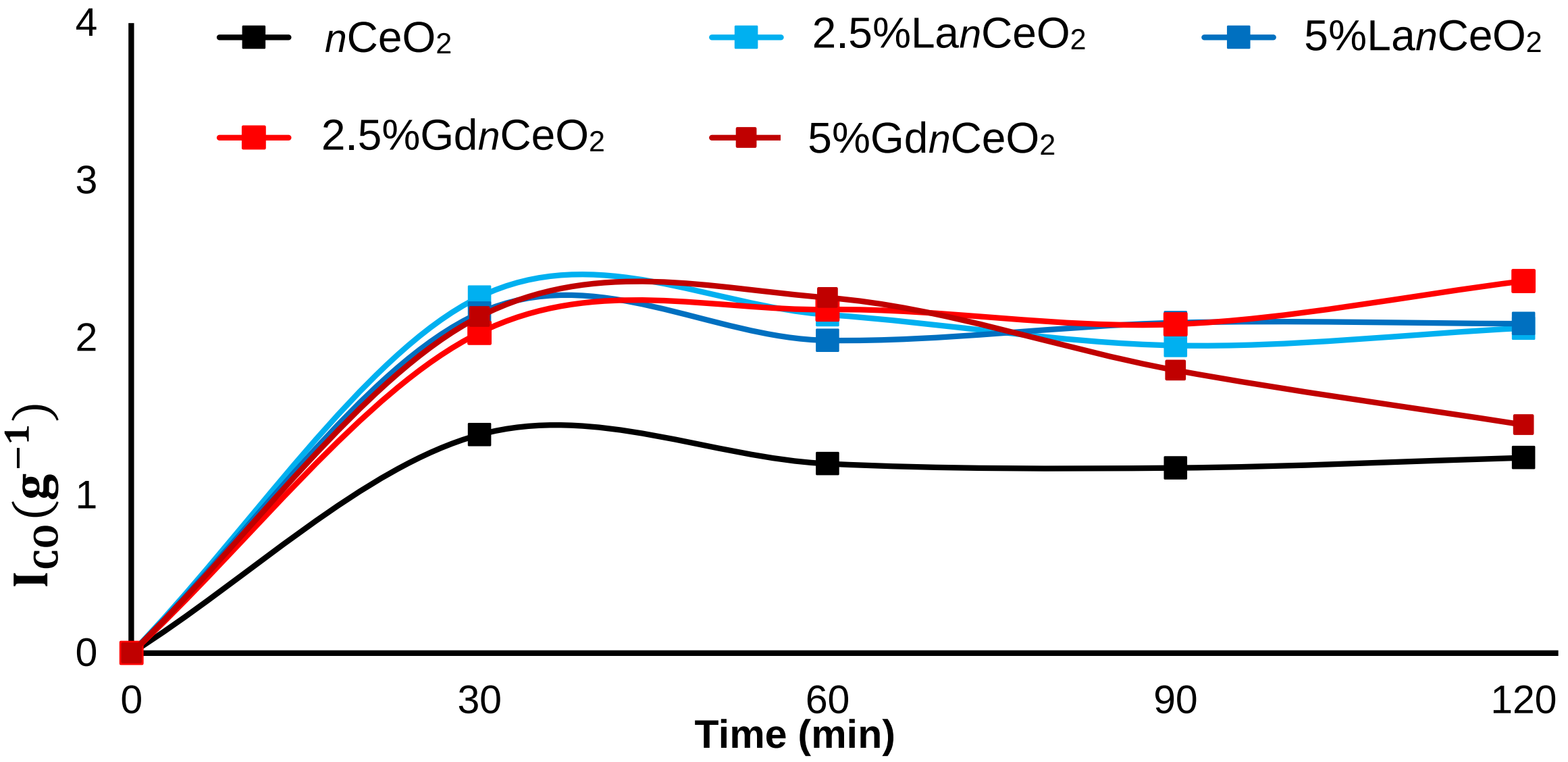

Figure S26: I_{CO} without applying potential onto different samples (*T*: 25 °C and *P*: ~1 bar).

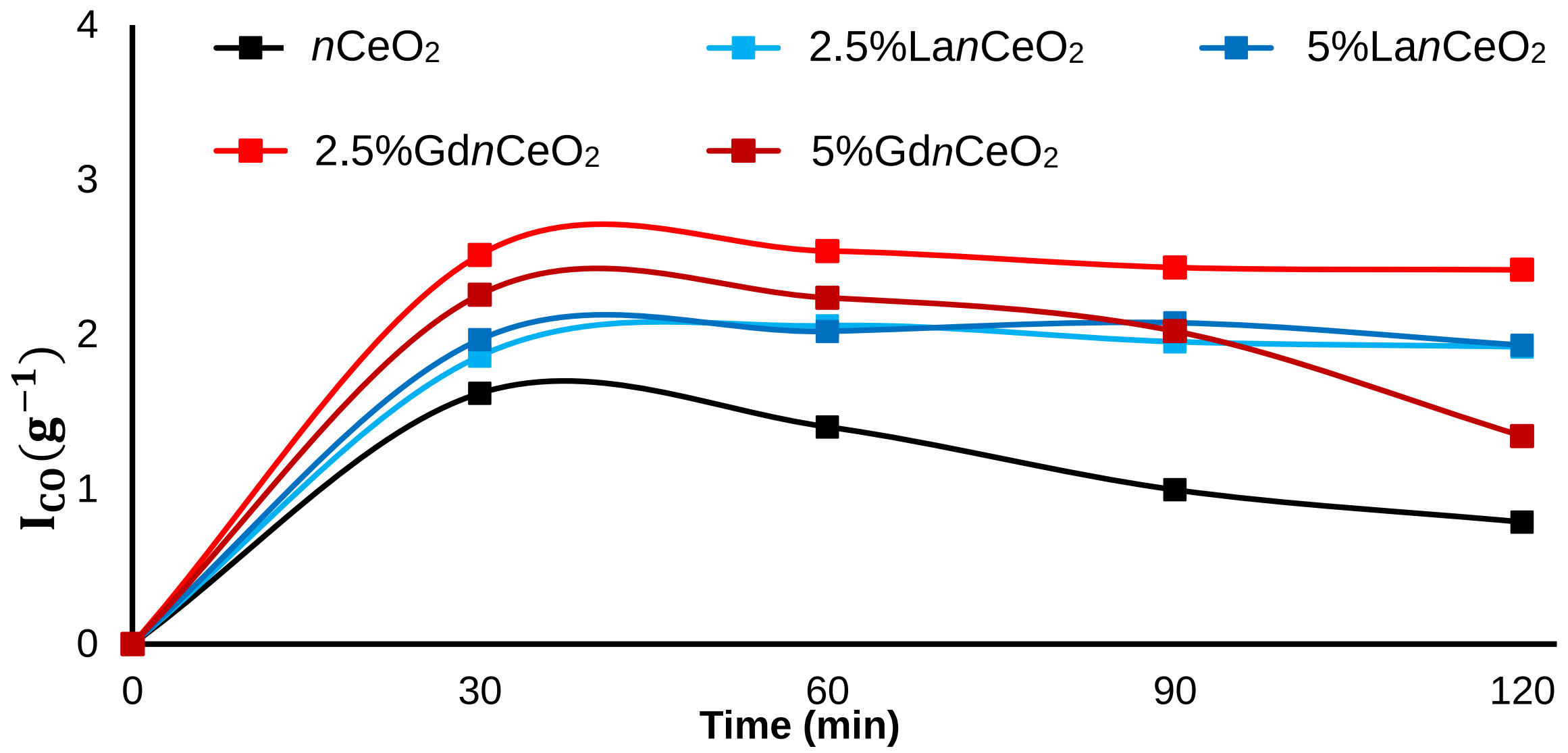

Figure S27: I_{CO} with applying potential onto different samples (*T*: 25 °C and *P*: ~1 bar).

Table S7 reports integration values related to presence of CO gas during the oxidation by different catalysts and without/with applying potential.

Table S7: Integration values related to CO gas for each measurement without and with applying potential (*T*: 25 °C and *P*: ~1 bar).

Sample	Initial integration	Integration after 30 min	Integration after 60 min	Integration after 90 min	Integration after 120 min
nCeO$_2$ without Potential	0.8933	0.6971	0.7345	0.7328	0.6976
	0.9266	0.7097	0.7401	0.7204	0.7299
	0.8806	0.6921	0.6836	0.7161	0.7178
nCeO$_2$ with Potential	0.7848	0.5475	0.6210	0.6256	0.6255
	0.6709	0.5535	0.5237	0.5599	0.5789
	0.7976	0.5372	0.5625	0.6345	0.6734
2.5%La^{3+}–nCeO$_2$ without Potential	0.7552	0.5186	0.5382	0.5816	0.5756
	0.7570	0.5023	0.4993	0.4991	0.4861
	0.7326	0.4619	0.4754	0.4852	0.4744
2.5%La^{3+}–nCeO$_2$ with Potential	0.7771	0.5228	0.4547	0.4954	0.4879
	0.7618	0.5318	0.5341	0.5326	0.5436
	0.7984	0.6070	0.6180	0.6076	0.6131
5%La^{3+}–nCeO$_2$ without Potential	0.7830	0.5470	0.5630	0.5550	0.5620
	0.7980	0.5470	0.5800	0.5620	0.5460
	0.7590	0.5170	0.5153	0.5110	0.5210
5%La^{3+}–nCeO$_2$ with Potential	0.7947	0.5294	0.5571	0.5666	0.5480
	0.8002	0.5852	0.5632	0.5449	0.6184
	0.7528	0.5524	0.5332	0.5271	0.5130
2.5%Gd^{3+}–nCeO$_2$ without Potential	0.7720	0.4780	0.5090	0.5080	0.5030
	0.7280	0.5190	0.4940	0.5300	0.4800
	0.7600	0.5560	0.5110	0.5000	0.4820
2.5%Gd^{3+}–nCeO$_2$ with Potential	0.7630	0.5160	0.4770	0.4760	0.4660
	0.7920	0.5050	0.5220	0.5050	0.5290
	0.7680	0.4450	0.4610	0.5080	0.4990
5%Gd^{3+}–nCeO$_2$ without Potential	0.7397	0.4990	0.4865	0.5477	0.5579
	0.7572	0.5099	0.5161	0.5391	0.5820
	0.7815	0.5683	0.5431	0.5766	0.6134
5%Gd^{3+}–nCeO$_2$ with Potential	0.7486	0.6137	0.5590	0.5783	0.6256
	0.8790	0.5769	0.6180	0.6500	0.6960
	0.9348	0.6393	0.6587	0.6620	0.7430

The average value and individual I_{CO} for each catalyst are written in Table S8 and Table S9, respectively.

Table S8: Average of I_{CO} without and with applying potential for each catalyst (*T*: 25 °C and *P*: ~1 bar).

**Sample**	I_{CO} **(g^{-1}) after 30 min**	I_{CO} **(g^{-1}) after 60 min**	I_{CO} **(g^{-1}) after 90 min**	I_{CO} **(g^{-1}) after 120 min**
nCeO$_2$ without Potential	1.3873 ± 0.1982	1.2031 ± 0.1917	1.1766 ± 0.2727	1.2412 ± 0.1872
nCeO$_2$ with Potential	1.6207 ± 0.5774	1.4036 ± 0.3290	0.9969 ± 0.1858	0.7883 ± 0.2538
2.5%La^{3+}–nCeO$_2$ without Potential	2.2603 ± 0.1866	2.1482 ± 0.2283	1.9525 ± 0.4260	2.0630 ± 0.4577
2.5%La^{3+}–nCeO$_2$ with Potential	1.8604 ±0.2988	2.0564 ± 0.6414	1.9529 ± 0.4136	1.9206 ± 0.4744
5%La^{3+}–nCeO$_2$ without Potential	2.1599 ± 0.0623	1.9861 ± 0.1754	2.0984 ± 0.1310	2.0922 ± 0.1338
5%La^{3+}–nCeO$_2$ with Potential	1.9679 ± 0.2799	2.0211 ± 0.0372	2.0767 ± 0.1183	1.9319 ± 0.3541
2.5%Gd^{3+}–nCeO$_2$ without Potential	2.0324 ± 0.4146	2.1807 ± 0.0758	2.0870 ± 0.2849	2.3613 ± 0.0931
2.5%Gd^{3+}–nCeO$_2$ with Potential	2.5153 ± 0.3309	2.5399 ± 0.1951	2.4334 ± 0.1290	2.4184 ± 0.1928
5%Gd^{3+}–nCeO$_2$ without Potential	2.1371 ± 0.2131	2.2579 ± 0.1271	1.7971 ± 0.1144	1.4503 ± 0.1000
5%Gd^{3+}–nCeO$_2$ with Potential	2.2585 ± 0.8964	2.2379 ± 0.4222	2.0226 ± 0.4644	1.3448 ± 0.3475

Table S9: I_{CO} values for each measurement without and with applying potential

(*T*: 25 °C and P: ~1 bar).

Sample	I_{CO} (g^{-1}) after 30 min	I_{CO} (g^{-1}) after 60 min	I_{CO} (g^{-1}) after 90 min	I_{CO} (g^{-1}) after 120 min
nCeO_2 without Potential	1.2570	0.9335	0.9482	1.2527
	1.6674	1.3621	1.5600	1.4645
	1.2375	1.3139	1.0216	1.0063
nCeO_2 with Potential	1.9241	1.1654	1.1179	1.1190
	0.8126	1.1767	0.7343	0.5022
	2.1255	1.8688	1.1383	0.7437
2.5%La^{3+}–*n*CeO_2 without Potential	2.0439	1.8282	1.3502	1.4166
	2.2374	2.2704	2.2726	2.4153
	2.4994	2.3459	2.2345	2.3573
2.5%La^{3+}–*n*CeO_2 with Potential	2.1712	2.8982	2.4637	2.5438
	1.9530	1.9279	1.9443	1.8243
	1.4570	1.3430	1.4508	1.3938
5%La^{3+}–*n*CeO_2 without Potential	2.0724	1.8932	1.9828	1.9044
	2.1954	1.8333	2.0308	2.2063
	2.2121	2.2318	2.2815	2.1658
5%La^{3+}–*n*CeO_2 with Potential	2.3627	2.0575	1.9528	2.1577
	1.7951	2.0358	2.2360	1.4319
	1.7458	1.9700	2.0413	2.2059
2.5%Gd^{3+}–*n*CeO_2 without Potential	2.6134	2.2801	2.2909	2.3446
	1.8100	2.0963	1.6841	2.2565
	1.6737	2.1657	2.2859	2.4827
2.5%Gd^{3+}–*n*CeO_2 with Potential	2.1347	2.5593	2.5702	2.6790
	2.4696	2.2918	2.4696	2.2186
	2.9415	2.7685	2.2604	2.3577
5%Gd^{3+}–*n*CeO_2 without Potential	2.2773	2.4272	1.6930	1.5706
	2.2981	2.2255	1.9564	1.4544
	1.8359	2.1210	1.7420	1.3258
5%Gd^{3+}–*n*CeO_2 with Potential	0.9950	1.6429	1.4143	0.8540
	2.9794	2.4919	2.1124	1.5668
	2.8011	2.5789	2.5411	1.6135

Results of I_{CO_2}

Figure S28 and S29 plot I_{CO_2} versus passing time of reaction under the condition of free potential and applied potential, respectively.

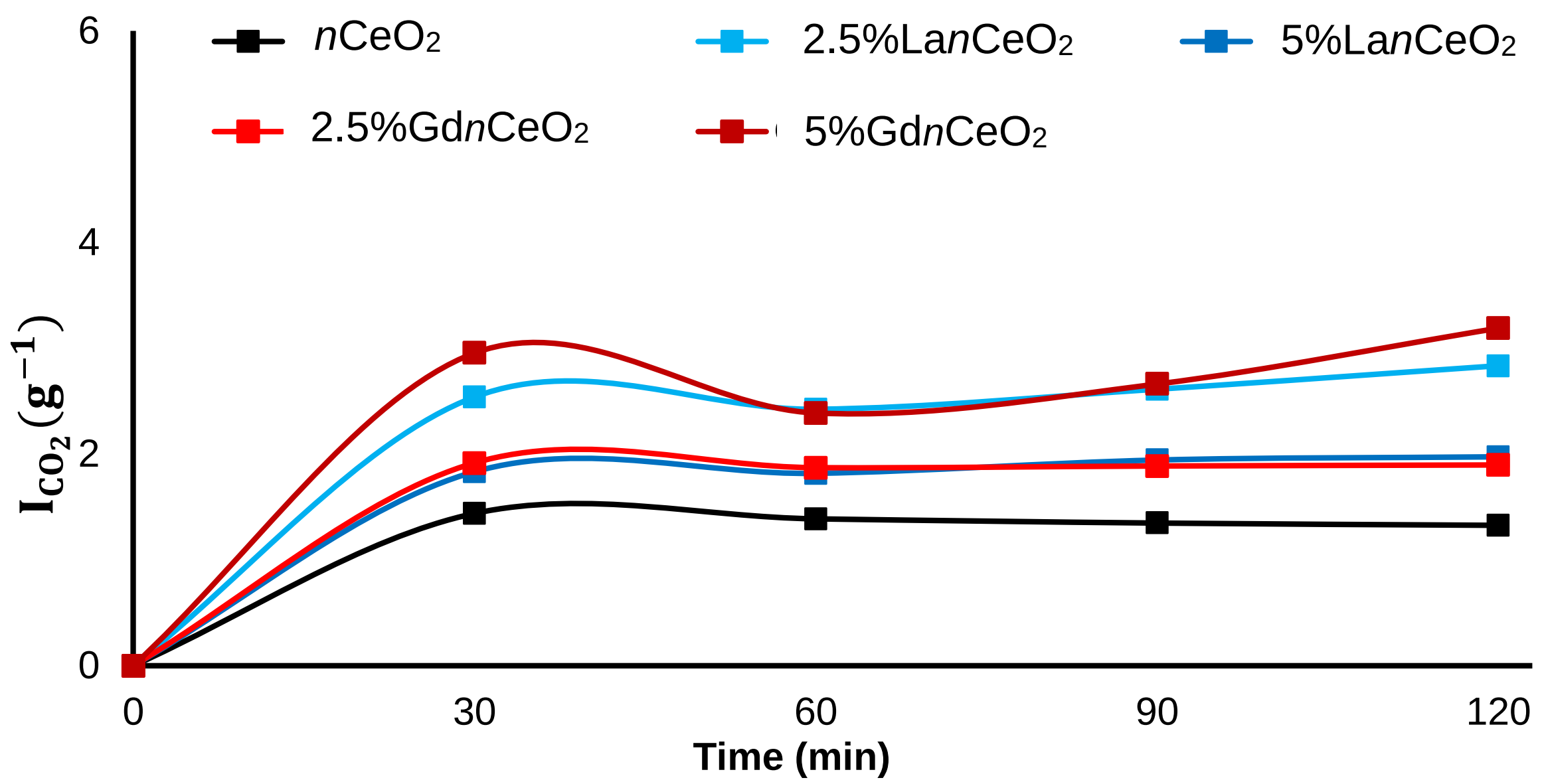

Figure S28: I_{CO_2} without applying potential (*T*: 25 °C and *P*: ~1 bar).

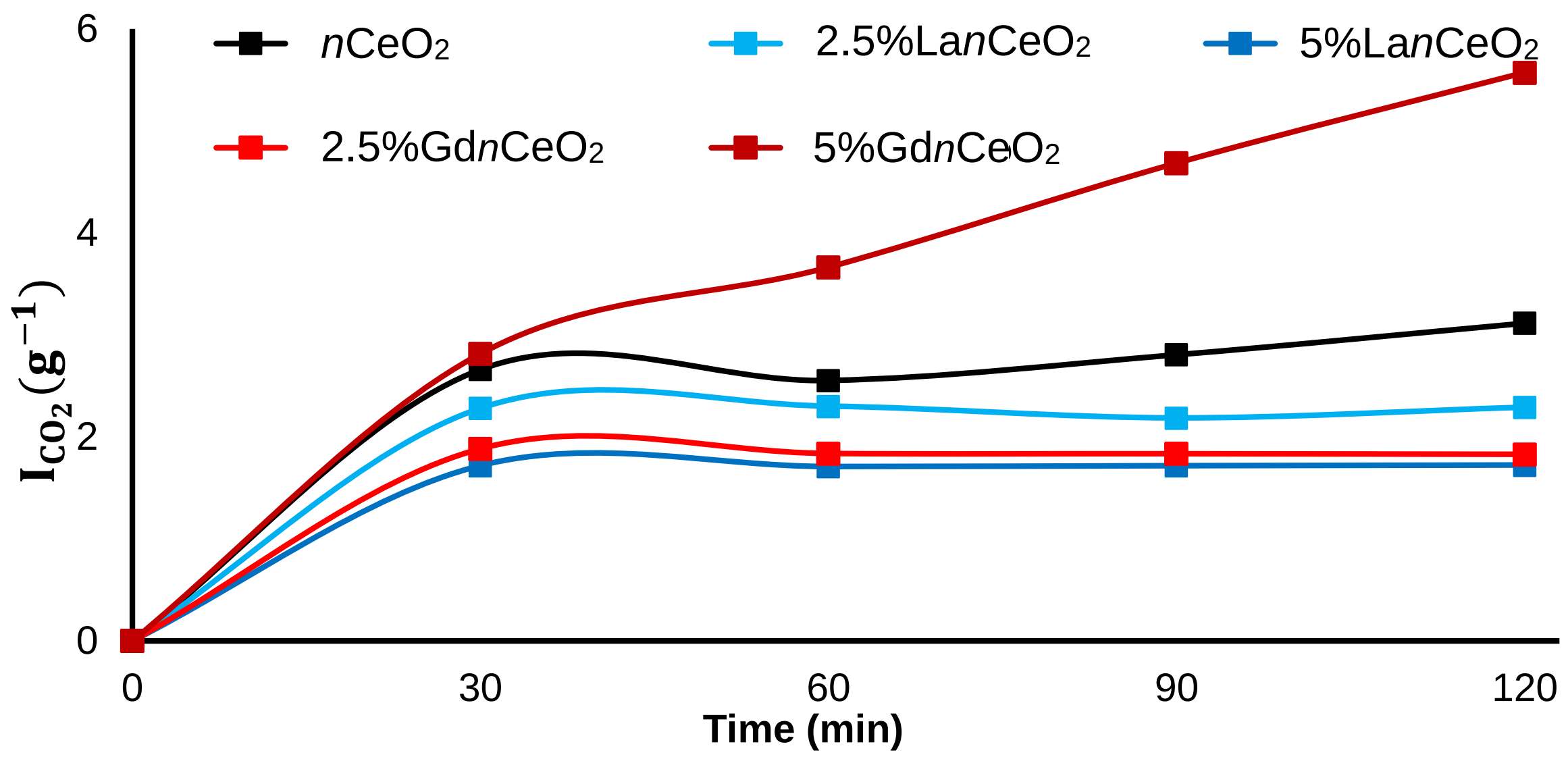

Figure S29: I_{CO_2} with applying potential (*T*: 25 °C and *P*: ~1 bar).

Table S10 reports integration values related to CO_2 gas produced by different catalysts and without/with applying potential.

Table S10: Integration values related to CO_2 gas for each measurement without and with applying potential (*T*: 25 °C and *P*: ~1 bar).

Sample	Initial integration	integration after 30 min	integration after 60 min	integration after 90 min	integration after 120 min
nCeO_2 without Potential	0.7450	0.6950	0.6207	0.5653	0.5561
	0.3826	0.6561	0.5490	0.4213	0.3618
	0.3962	0.6362	0.4878	0.4136	0.3446
nCeO_2 with Potential	1.3570	3.8790	3.6000	4.3380	4.6410
	1.2730	2.0330	2.1570	2.2050	2.6800
	1.0220	3.1440	2.6720	3.2910	4.1620
2.5%La^{3+}–*n*CeO_2 without Potential	-0.0320	0.7130	0.5100	1.1590	1.4730
	0.0700	1.1720	0.9250	0.9340	1.6470
	0.1460	0.7740	0.7230	0.7260	0.7020
2.5%La^{3+}–*n*CeO_2 with Potential	0.0840	0.7530	0.7120	0.6100	0.7450
	0.1070	0.5180	0.4660	0.4590	0.5390
	0.0880	2.5460	2.7550	2.2330	2.5880
5%La^{3+}–*n*CeO_2 without Potential	0.3260	0.1960	0.1980	0.2350	0.2240
	0.1950	0.0950	0.1010	0.0990	0.1080
	0.1610	0.9200	0.8370	1.4280	1.5810
5%La^{3+}–*n*CeO_2 with Potential	-0.0210	-0.0050	-0.0260	-0.0270	-0.0090
	0.0450	0.0200	0.0240	-0.0010	0.0050
	0.0850	0.0790	0.0510	0.1140	0.1250
2.5%Gd^{3+}–*n*CeO_2 without Potential	0.1430	0.5950	0.4730	0.5660	0.6600
	0.1390	0.6120	0.4420	0.4790	0.4690
	0.1370	0.3600	0.4210	0.3730	0.3450
2.5%Gd^{3+}–*n*CeO_2 with Potential	0.0520	0.5230	0.4230	0.4320	0.4290
	-0.0450	0.2520	0.2540	0.2210	0.2410
	0.0230	0.5380	0.3900	0.4000	0.3540
5%Gd^{3+}–*n*CeO_2 without Potential	0.2950	0.8750	0.9270	1.1910	1.7290
	-0.0430	1.1930	1.2490	1.7200	2.7650
	0.0920	4.1220	1.1290	1.7520	2.7840
5%Gd^{3+}–*n*CeO_2 with Potential	0.0160	2.7550	4.0810	4.8640	5.2490
	0.9050	3.0010	4.4480	7.0600	9.2480
	0.2818	2.0732	4.0592	6.5500	9.1840

The average value and individual I_{CO_2} for each catalyst are written in Table S11 and Table S12, respectively.

Table S11: Average of I_{CO_2} without and with applying potential for each catalyst (*T*: 25 °C and *P*: ~1 bar).

**Sample**	I_{CO_2} **(g^{-1}) after 30 min**	I_{CO_2} **(g^{-1}) after 60 min**	I_{CO_2} **(g^{-1}) after 90 min**	I_{CO_2} **(g^{-1}) after 120 min**
nCeO$_2$ without Potential	1.4409 ± 0.0653	1.3896 ± 0.0511	1.3499 ± 0.0466	1.3286 ± 0.0356
nCeO$_2$ with Potential	2.6612 ± 0.2386	2.5541 ± 0.1688	2.8062 ± 0.2963	3.1155 ± 0.2486
2.5%La^{3+}–nCeO$_2$ without Potential	2.5404 ± 0.4645	2.4246 ± 0.4166	2.6140 ± 0.6792	2.8361 ± 0.7435
2.5%La^{3+}–nCeO$_2$ with Potential	2.2800 ± 0.4560	2.3003 ± 0.5217	2.1858 ± 0.4015	2.2901 ± 0.4644
5%La^{3+}–nCeO$_2$ without Potential	1.8343 ± 0.2833	1.8191 ± 0.2586	1.9450 ± 0.4223	1.9754 ± 0.4661
5%La^{3+}–nCeO$_2$ with Potential	1.7175 ± 0.0520	1.7085 ± 0.0451	1.7164 ± 0.0674	1.7232 ± 0.0630
2.5%Gd^{3+}–nCeO$_2$ without Potential	1.9185 ± 0.0962	1.8707 ± 0.0475	1.8863 ± 0.0603	1.8966 ± 0.0713
2.5%Gd^{3+}–nCeO$_2$ with Potential	1.8849 ± 0.0834	1.8385 ± 0.0500	1.8360 ± 0.0610	1.8305 ± 0.0515
5%Gd^{3+}–nCeO$_2$ without Potential	2.9569 ± 0.8316	2.3899 ± 0.1450	2.6636 ± 0.1955	3.1920 ± 0.3289
5%Gd^{3+}–nCeO$_2$ with Potential	2.8148 ± 0.5091	3.6627 ± 0.4748	4.6831 ± 0.1369	5.5712 ± 0.3678

Table S12: I_{CO_2} values for each measurement without and with applying potential (*T*: 25 °C and *P*: ~1 bar).

Sample	I_{CO_2} (g^{-1}) after 30 min	I_{CO_2} (g^{-1}) after 60 min	I_{CO_2} (g^{-1}) after 90 min	I_{CO_2} (g^{-1}) after 120 min
nCeO_2 without Potential	1.3564	1.3215	1.2955	1.2912
	1.4510	1.4026	1.3449	1.3180
	1.5154	1.4447	1.4093	1.3764
nCeO_2 with Potential	2.9432	2.7928	3.1906	3.3539
	2.3597	2.4388	2.4694	2.7725
	2.6808	2.4308	2.7587	3.2201
2.5%La^{3+}–*n*CeO_2 without Potential	3.1873	3.0116	3.5732	3.8448
	2.3162	2.1747	2.1798	2.5885
	2.1177	2.0874	2.0892	2.0749
2.5%La^{3+}–*n*CeO_2 with Potential	2.0108	1.9879	1.9311	2.0063
	1.9072	1.8776	1.8736	1.9191
	2.9222	3.0353	2.7527	2.9449
5%La^{3+}–*n*CeO_2 without Potential	1.6419	1.6431	1.6647	1.6583
	1.6261	1.6296	1.6284	1.6336
	2.2349	2.1847	2.5419	2.6344
5%La^{3+}–*n*CeO_2 with Potential	1.7001	1.6880	1.6875	1.6978
	1.6642	1.6665	1.6522	1.6556
	1.7882	1.7711	1.8092	1.8162
2.5%Gd^{3+}–*n*CeO_2 without Potential	1.9032	1.8348	1.8870	1.9397
	2.0394	1.9378	1.9599	1.9540
	1.8047	1.8395	1.8121	1.7961
2.5%Gd^{3+}–*n*CeO_2 with Potential	1.9381	1.8812	1.8863	1.8846
	1.7672	1.7683	1.7503	1.7612
	1.9494	1.8659	1.8715	1.8455
5%Gd^{3+}–*n*CeO_2 without Potential	2.2044	2.2369	2.4023	2.7394
	2.5505	2.5847	2.8725	3.5109
	4.1160	2.2382	2.7161	3.3257
5%Gd^{3+}–*n*CeO_2 with Potential	3.5116	4.3317	4.8159	5.0541
	2.6235	3.3775	4.7386	5.8787
	2.3092	3.2788	4.4948	5.7807

References

1. Heinschke, S.; Schneider, J. J. *Adv. Sci.* **2025**, *12*, e04617. doi:10.1002/advs.202504617
2. Basak, M.; Rahman, Md. L.; Ahmed, Md. F.; Biswas, B.; Sharmin, N. *J. Alloy. Compd.* **2022**, *895*, 162694. doi:10.1016/j.jallcom.2021.162694
